# Phytolith evidence for the pastoral origins of multi-cropping in Mesopotamia (ancient Iraq)

**DOI:** 10.1038/s41598-021-03552-w

**Published:** 2022-01-10

**Authors:** Elise Jakoby Laugier, Jesse Casana, Dan Cabanes

**Affiliations:** 1 Graduate Program in Ecology, Evolution, Environment, and Society (EEES), Dartmouth College, Hanover, NH 03755 USA; 2 Department of Anthropology, Dartmouth College, Hanover, NH 03755 USA; 3 Department of Anthropology, Rutgers University, New Brunswick, NJ 08901 USA; 4 Center for Human Evolutionary Studies (CHES), Rutgers University, New Brunswick, NJ 08901 USA

**Keywords:** Archaeology, Anthropology

## Abstract

Multi-cropping was vital for provisioning large population centers across ancient Eurasia. In Southwest Asia, multi-cropping, in which grain, fodder, or forage could be reliably cultivated during dry summer months, only became possible with the translocation of summer grains, like millet, from Africa and East Asia. Despite some textual sources suggesting millet cultivation as early as the third millennium BCE, the absence of robust archaeobotanical evidence for millet in semi-arid Mesopotamia (ancient Iraq) has led most archaeologists to conclude that millet was only grown in the region after the mid-first millennium BCE introduction of massive, state-sponsored irrigation systems. Here, we present the earliest micro-botanical evidence of the summer grain broomcorn millet (*Panicum miliaceum*) in Mesopotamia, identified using phytoliths in dung-rich sediments from Khani Masi, a mid-second millennium BCE site located in northern Iraq. Taphonomic factors associated with the region’s agro-pastoral systems have likely made millet challenging to recognize using conventional macrobotanical analyses, and millet may therefore have been more widespread and cultivated much earlier in Mesopotamia than is currently recognized. The evidence for pastoral-related multi-cropping in Bronze Age Mesopotamia provides an antecedent to first millennium BCE agricultural intensification and ties Mesopotamia into our rapidly evolving understanding of early Eurasian food globalization.

## Introduction

Multi-cropping, defined here as the seasonally sequential production of multiple crops on the same land in the same year, is an agricultural technique aimed at diversifying and intensifying economic and subsistence-based food, fodder, and forage production^1–3^. Similar to the contemporary world, this form of agricultural production was vital for provisioning large urban centers and financing imperial ambitions across ancient Eurasia^4^. For millennia, grain production in western Eurasia was limited to winter cereals, primarily wheat and barley, both of which were locally domesticated and adapted to Southwest Asia’s Mediterranean climate, with cool, wet winters and hot, dry summers. Summer grains and their wild relatives are not native to the region and thus summer cultivation only became possible with the translocation of millets and other East Asian and African domesticates.

**Figure 1:**
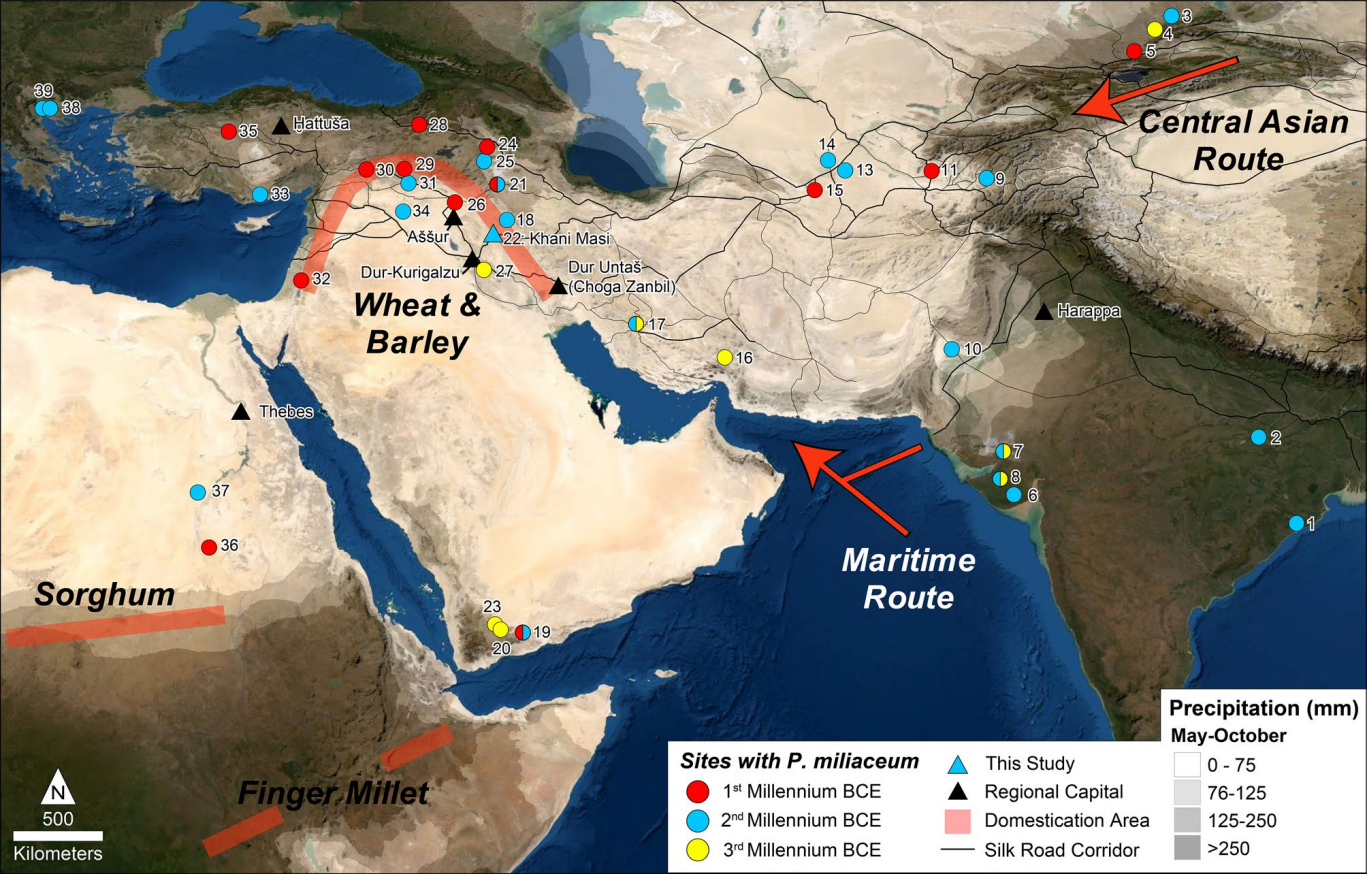
Map of archaeological sites with archaeobotanical evidence for broomcorn millet (*Panicum miliaceum*) from the 3rd–1st millennium BCE. See Supplementary Table S2 for site data sources. Summer precipitation (May–October)^23^ is displayed in grayscale (after^5^). Red lines and arrows indicate domestication areas and translocation routes (after^13,19^), and black lines indicate later silk road corridors (after^24^). This figure was generated in Esri’s ArcGIS 10.6.1 (http://www.esri.com/software/arcgis) using Esri World Imagery (Sources: Esri, DigitalGlobe, GeoEye, i-cubed, USDA FSA, USGS, AEX, Getmapping, Aerogrid, IGN, IGP, swisstopo, and the GIS User Community).

Millets represent a variety of fast-growing, small-seeded summer grains, initially domesticated in both Africa and northern China. Millets require summer rainfall (May–October > 120 mm) or irrigation^5,6^. Their short life cycle, drought tolerance, minimal maintenance, high returns, and protein-rich grains make millets versatile, nutritious, and labor-effective food sources for both people and livestock^7–9^. Two of the East Asian millets, broomcorn (*Panicum miliaceum*) and foxtail (*Setaria italica*) millet, were likely transported to western Eurasia both across the continent through Central Asia and along maritime trade routes (Fig. 1)^5,10^. Broomcorn (*P. miliaceum*), in particular, would eventually become one of the most important cereal grains in ancient Eurasia^4^.

By the mid-second millennium BCE, long-distance exchange networks connected all of Eurasia marking the near completion of the “Trans-Eurasian Exchange” in which East Asian domesticates arrive in Southwest Asia and Europe and wheat and barley reach East Asia^11–14^. Although domesticated millet is found throughout Central and South Asia and as far west as eastern Europe, cultivation of the crop is thought to be mainly restricted to areas with sufficient summer precipitation (Fig. 1)^5^. Even today broomcorn millet (*Panicum miliaceum*) is a very minor cultivar in Iraq^15,16^ and rarely grows ferally even in perennially damp places^17^. As a result, most archaeologists believe millet was only introduced to Mesopotamia (ancient Iraq) and other areas that lack summer precipitation with the construction of massive, state-sponsored irrigation systems during the mid-first millennium BCE, which would have made multi-cropping possible and worthwhile^5,18^. In contrast, textual evidence suggests millet may have been cultivated in Mesopotamia as early as the third millennium BCE, possibly being introduced via maritime routes from the Indus Valley^10,19^ or overland via expanding Bronze Age trade networks^20–22^.

### Previous evidence for millet cultivation in Mesopotamia

#### Textual evidence

Mesopotamian textual specialists have long argued that millet was an important crop in the region as early as the third millennium BCE^25,26^. The Akkadian term for millet (*duḫnu*/*tuḫnu*) is first explicitly mentioned in mid-second millennium BCE cuneiform texts, found at the Mesopotamian sites of Nippur and Nuzi^27^. Texts from Nuzi suggest that millet was planted in conjunction with sesame: *“plant sesame and millet! there is one homer of sesame and millet which is already planted,*” or gifted as grain along with barley: “*PN [personal name] gave to PN*_*2*_*, PN*_*3*_*, and PN*_*4*_*, four homers of barley and two homers of millet as…for the properties*^27^.” Some scholars link *duḫnu*/*tuḫnu* to the Akkadian word *arsikku* (Sumerian: *ar zig*), possibly pushing references to millet back into the third millennium BCE^26,28^. Old Akkadian (third millennium BCE) texts also refer generically to both “early grain” (Sumerian: *še nim*; Akkadian: *harpu*) and “late grain” (Sumerian: *še sig*; Akkadian: *uppulu*), which was sown in spring and harvested in late summer and thus may refer to either millet or sesame^26,29,30^. These are the only known summer-sown “grains,” and certainly all the technology needed for their cultivation would have been available long before the second millennium BCE^31^.

#### Archaeobotanical evidence

Textual evidence contrasts sharply with archaeological perspectives that largely ignore the possibility or implications of millet multi-cropping in Mesopotamia prior to the first millennium BCE. Some archaeologists do not doubt that millet was also present in semi-arid Mesopotamia during the second millennium BCE because it is contemporaneously present in adjacent areas with more temperate climates^32^, and sesame, also a summer crop, is a known cultivar in the region from the third millennium BCE onwards^6,33,34^. However, millet is rarely mentioned in discussions of early Mesopotamian agriculture^34,35^ and is generally excluded from models of Bronze Age Mesopotamian food production^36,37^.

The contradiction between textual and archaeological perspectives is due to the nearly complete absence of archaeobotanical evidence for millet in Iraq in all periods^38^ and, until recently, its general scarcity in adjacent regions. The earliest unequivocal archaeobotanical evidence for broomcorn millet in Mesopotamia is from c. 700 BCE when millet grains are found in large numbers at Nimrud and Fort Shalmenesar (Fig. 1, no. 26; Supplementary Table S2)^39^. Citing Boserup^40^, Miller et al.^5^ argue that millet may have been known in Mesopotamia, but was absent due to ecological constraints (i.e., the lack of summer precipitation) and it was never used prior to the large-scale Neo-Assyrian imperial intensification systems, even as a diversification or risk reduction strategy (see also^41^). Likewise, Rosenzweig^18^ credits the Neo-Assyrians and their agricultural maximization policies with the introduction of millet and other non-local crops to northern Mesopotamia.

However, archaeobotanical remains are not entirely absent prior to the first millennium BCE. Earlier evidence for *Panicum miliaceum* may be present in impressions of millet grains on ceramics from the site of Jemdet Nasr (ancient Kish) in southern Iraq dating to 3000 BCE^42,43^, but the interpretation of botanical impressions has been argued to be unreliable^44^. A few charred *Panicum* grains were reportedly found inside a small jar from the same site^45^, and a single grain of *P. miliaceum* was identified from a secure Late Bronze Age (14th–13th cent. BCE) oven context at Gurga Chiya in the Shahrizor plain in northeastern Iraq^46^. In northern Syria, isolated grains of *P. miliaceum* are also reported in second millennium BCE contexts at Tell Sheikh Hamad^47,48^ and Tell Mozan in northeast Syria^49^; although the Tell Mozan finds are not mentioned in the final report^50^. The rarity of millets in archaeobotanical data from Bronze Age Mesopotamia has led archaeologists to interpret these finds as exotic imports, intrusive grains, or very minor cultivars, and thus millet has played almost no role in our interpretations of agro-pastoral production in the region.

### Second millennium BCE Khani Masi

**Figure 2:**
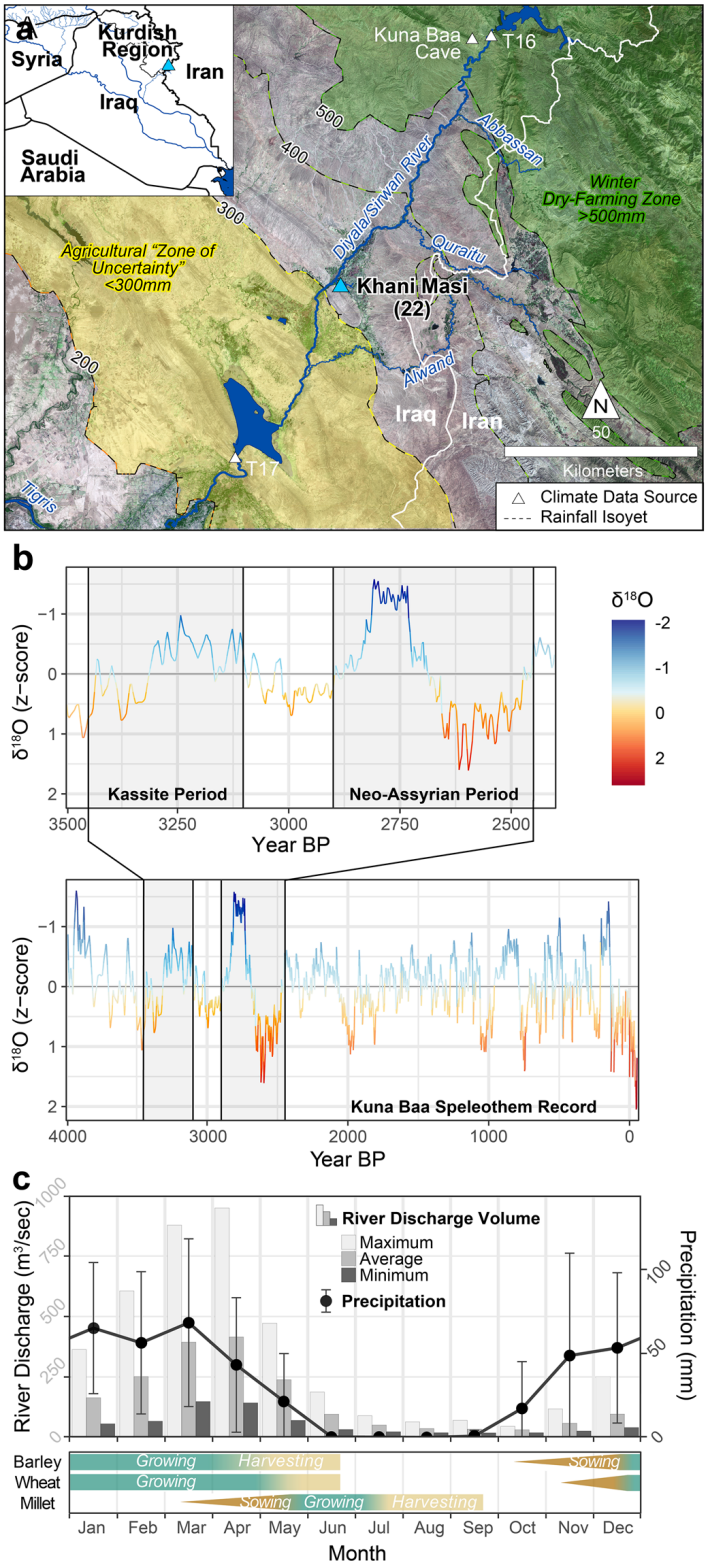
The Khani Masi region and its environmental context. (**a**) Map of Upper Diyala/Sirwan River region with perennial water sources, growing season rainfall isohyets (November–April)^23^, agricultural zones^59^, and climate proxy sources indicated. This figure was generated in Esri’s ArcGIS 10.6.1 (http://www.esri.com/software/arcgis) using Landsat 8 imagery (4 Oct 2021; courtesy USGS and NASA). (**b**) Paleoclimate speleothem record of the last c. 4000 years from the Kuna Ba cave (Kurdistan Region, Iraq; **a**)^56^ highlighting the Kassite (c. 1550–1150 BCE) and Neo-Assyrian periods (c. 10th–early seventh centuries BCE). (**c**) Averaged monthly Diyala/Sirwan River discharge volume from meter stations north (T16; **a**) and south (T17; Fig. 2A) of Khani Masi for the years 1931–1955, prior to the Darbandikhan and Hamrin dam constructions^60^. The black line indicates average (± SD) precipitation by month from 1960 to 2016^57^ compared with the agricultural cycles of major crop types by month for Iraq^16^.

This study presents new data from the site of Khani Masi, located along the Upper Diyala/Sirwan River, a tributary of the Tigris River, in the Kurdistan Region of Iraq (Fig. 2a). Khani Masi is composed of more than a dozen mounds clustered along a relict levee above the Diyala/Sirwan River. From 2014 to 2019, the Sirwan Regional Project (SRP) initiated a program of archaeological investigations focusing on large-scale excavation of a sprawling low mound (SRP 46), which measures c. 5 ha in area and was occupied exclusively during the mid- to late-second millennium BCE^51–54^. At this time, Khani Masi appears to have close cultural and economic ties to Kassite Babylonia, centered in southern Mesopotamia. Excavations have revealed a sequence of major construction episodes, with the earliest phases dated to around 1450 BCE and subsequent building phases during the fourteenth and thirteenth centuries BCE. Settlement at SRP 46 ended following the abandonment of an extensive baked mudbrick building complex around 1100 BCE, and there is no evidence that this part of the site was ever reoccupied^51^.

The region in which Khani Masi is located has a typical Mesopotamian steppe climate (Irano-Turanian vegetation), with cool, wet winters and hot, dry summers^55^, and regional paleoclimate data suggest that a similar climate system prevailed during the second millennium BCE (Fig. 2b)^56^. Today, average winter rainfall in the Khani Masi area is marginally sufficient for dry-farmed wheat and barley cultivation (Nov–April 1970–2000: 314 ± 51 mm^23^; 334.6 ± 115.3 mm^57^) (Fig. 2c). The high interannual variability in precipitation means that today, and probably during historic periods, irrigation was necessary to support reliable agriculture, even for the Southwest Asian crops, wheat and barley. From May through October, Khani Masi only receives an average of 17 ± 9 mm (1970–2000)^23^ and thus any summer cultivation would unquestionably require irrigation. Although direct evidence for irrigation works predating the first millennium CE have not yet been observed in the region, the area surrounding Khani Masi (a Kurdish name meaning “spring of the fishes”), is replete with perennial, spring-fed streams, supplied by groundwater originating in the Zagros Mountains to the northeast. This well-watered plain has a rich history of human occupation dating back to the Neolithic period^58^, and thus it is reasonable to conclude that irrigation was likely practiced for many millennia.

**Figure 3:**
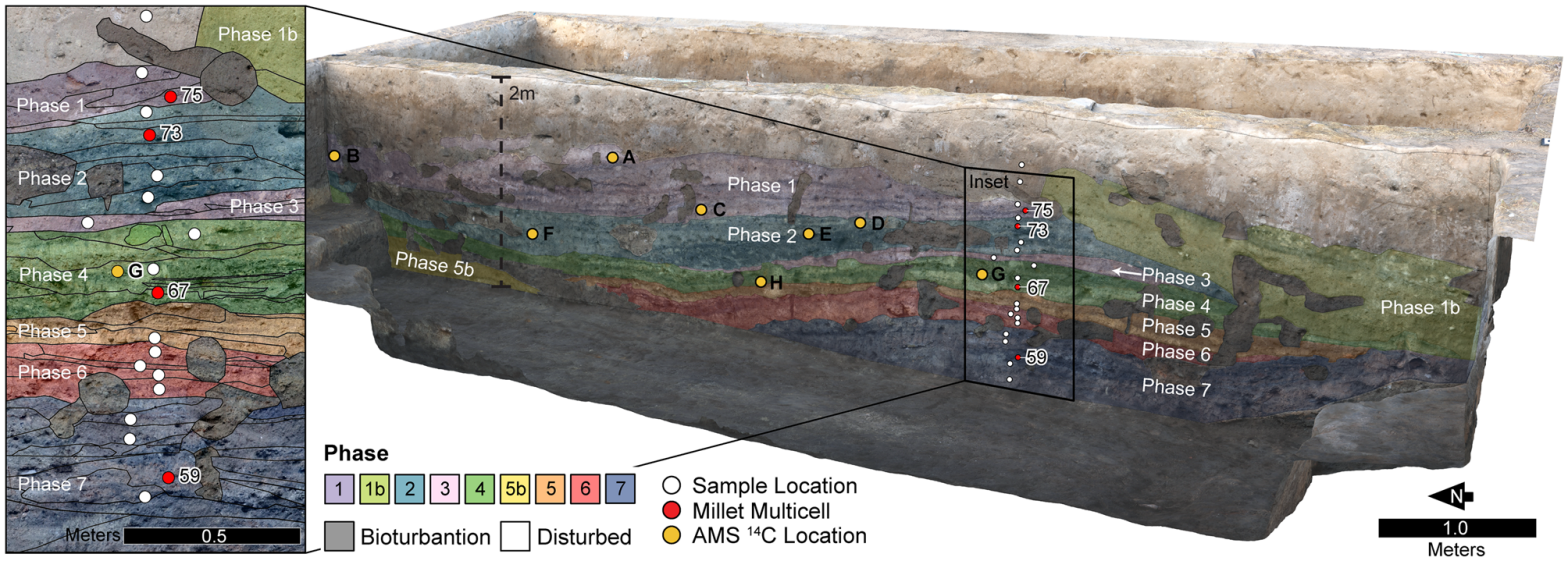
Eastern profile of Khani Masi Trench Y82. Colors represent major stratigraphic phases, or depositional episodes. Circles indicate sediment sample locations and approximate locations of excavated charcoal samples (Supplementary Tables S1 and S3). Samples with measurable interdigitating phytoliths in anatomical connection (silica skeletons) are highlighted in red. Figure modified from^54^. This figure was generated using Agisoft’s Metashape Professional Software v. 1.5.3 (http://www.agisoft.com/).

**Figure 4:**
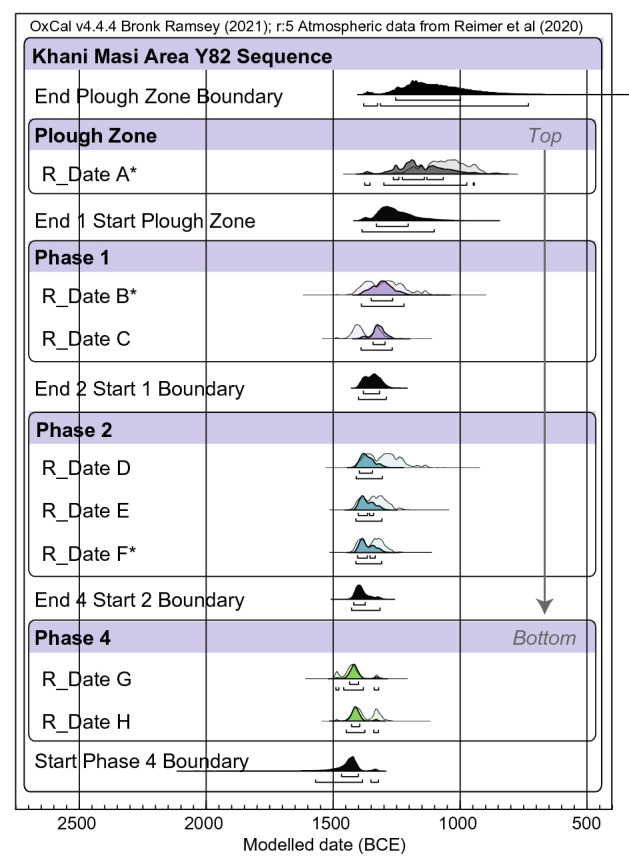
OxCal^65^ multiplot of Bayesian modelled ^14^C dates by phase (indices: Amodel 100.1, Aoverall 100.9). The modeled start date for phase 4, stratigraphically earlier than sample 59, is 1571–1322 BCE (± 2σ, 95.4% confidence) (Supplementary Tables S3–S4). Asterisk denotes new dates for this study. Unmodelled dates were previously reported by Laugier et al.^54^.

In 2019, the SRP excavations near the center of the site uncovered what appear to be a large, deep midden deposit (Trench Y82, Fig. 3). Excavations of midden deposits are not uncommon in the greater ancient Near East (e.g.^61,62^), but have rarely been studied in detail in Mesopotamia^54,63,64^. Abundant ceramics and AMS carbon-14 samples securely date the deposit to the mid-late second millennium BCE (Figs. 3, 4 and Supplementary Tables S3–4). During phytolith morphological analysis of the sediments from Trench Y82^54^ (see Supplementary Text), we encountered phytoliths in anatomical connection, or multicellular structures (also known as silica skeletons^66^ or articulated groups^67^), composed of interdigitating phytolith morphotypes^68,69^ similar to *Panicum miliaceum* in ten samples (Table S1). In general, phytolith preservation was good across all samples (Supplementary Text) as is typical for archaeological sites (or tells) in Southwest Asia where phytoliths are removed from the silica cycle^70–73^. No interdigitating phytolith morphotypes distinctive of domesticated *Panicum* sp. were observed in surface control samples, and millet is not currently cultivated in the area. Thus, modern contamination should be excluded.

Independent of the analyses performed by Laugier et al.^54^ (supplementary text, Table S1, and Figure S1), in this study, we performed a morphometric analysis on 30 multicellular structures composed exclusively of interdigitating phytoliths from four samples (Fig. 3) to determine their taxonomic origin (“Material and Methods” section). interdigitating phytoliths in anatomical connection were abundant and easily photographed in sample 59 (*N* = 27), while samples 67, 73, and 75 yielded only a single interdigitating multicellular structure each. Providing context to these four samples, samples 59, 73, and 75 are from burned animal dung-rich deposits while sample 67 is from an outdoor surface (Supplementary Text and Table S1). Sample 59 is the earliest dung-rich sediment in the Trench Y82 sequence, and post-depositional organic decay is indicated by the presence of authigenic phosphate^54^. Phytolith morphotype results showed that riparian vegetation like sedges (Cyperaceae) were rare (< 2.1%) (Supplementary Text and Fig. S1). bilobate short cells, distinctive of Panicoideae (C_4_) vegetation like millet, represent 30% of the short cell assemblage in sample 59 (the most of any sample at Khani Masi) and between 9.3 and 10.4% in samples 67, 73, and 75 (Supplementary Text and Fig. S1)^54^.

Although some scholars argue that morphometric analysis is not necessary to securely differentiate *Panicum miliaceum* inflorescence bracts (upper lemma and palea) from other domesticated millets^74^, *P. miliaceum* and other domesticated millets share several features with their wild relatives and other weedy Panicoideae (C_4_) grasses^75,76^. For example, the *Panicum* species, *Panicum bisulcatum* (Japanese panicgrass) and *Panicum repens* (torpedo grass), are morphometrically very similar to *P. miliaceum* and can only be distinguished from broomcorn millet based on a single criterion^77,78^. *P. bisulcatum* is not native to Iraq, but *P. repens* is native and present in riparian areas^17,79,80^. In fact, five of the nine genera representing millets and their wild relatives^81^ are present in modern Iraq (*Digitaria*, *Echinochloa, Panicum*, *Paspalum*, *Setaria*, and *Sorghum*) (Supplementary Table S6)^17,80^. Thus, it is necessary to use at least five diagnostic criteria to ensure secure species-level identifications of millet inflorescence phytoliths (Supplementary Table S5)^68,75–77,82^. Like phytoliths from grass inflorescences, bilobate short cells can be used to distinguish between domesticated millet species^83^. However, because the primary concern in this study was differentiating *P. miliaceum* from wild panicoids, different bilobate short cell types were not analyzed here.

Using five criteria, *Panicum miliaceum* inflorescence phytoliths can be confidently differentiated from all other known millet-like panicoideae species native to Iraq (Supplementary Fig. S2 and Table S5–6)^68,76–78,82–86^. That is, based on the current knowledge of phytolith morphometrics and the native ranges of species that produce interdigitating phytolith morphotypes*, P. miliaceum* phytoliths are distinct from all other known phytolith reference species except *Panicum miliaceum L. subsp. Ruderale* (Kitag.) Tzvelev, the debated progenitor, feral relative, or weedy companion of domesticated *P. miliaceum*^78^. *Panicum ruderale* is not native to Iraq and thus its presence would also indicate human translocation.

## Results

**Figure 5:**
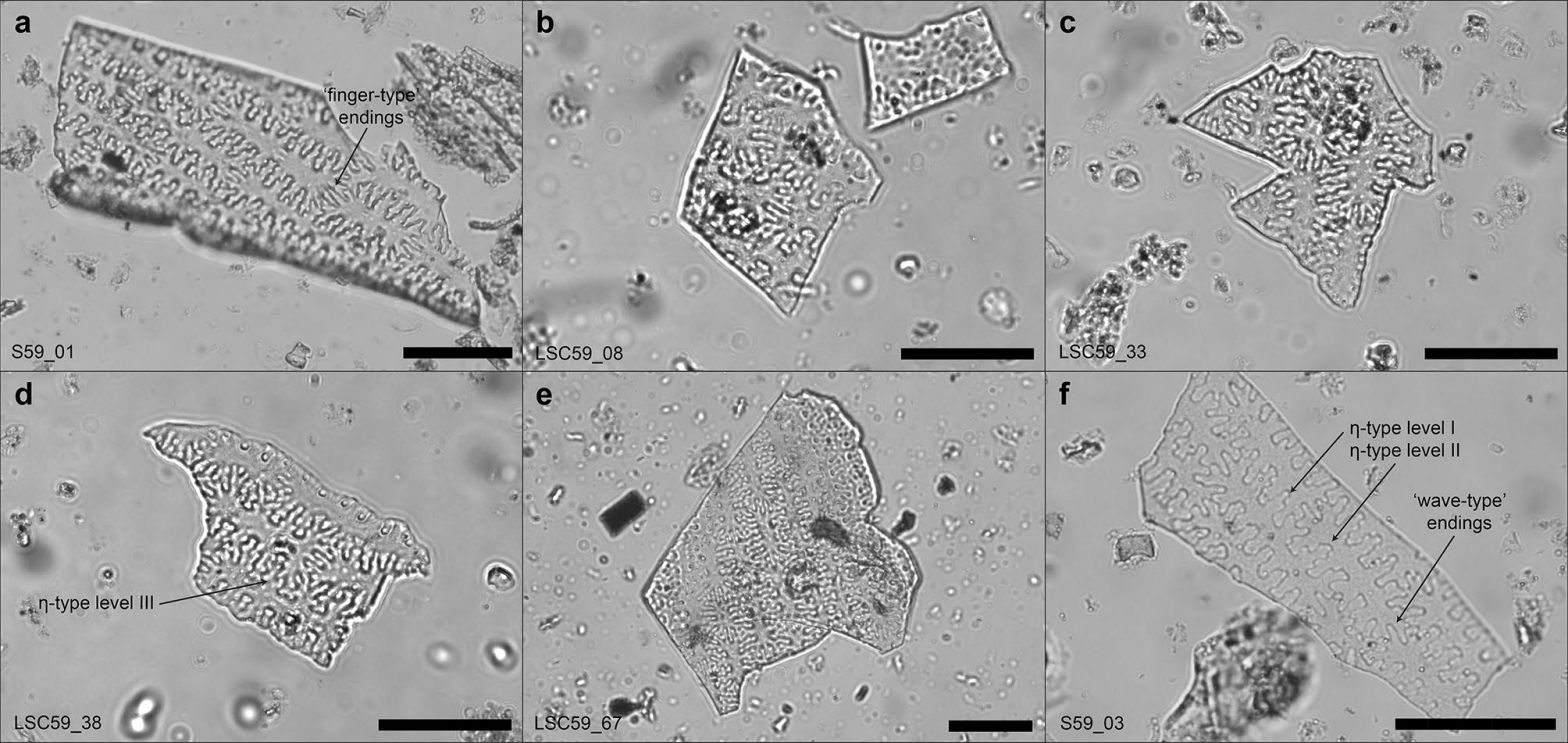
Microscope images of a selection of measured interdigitating multicellular phytoliths. (**a**–**e**) interdigitating multicellular structures from sample 59 that contain long cells (elongate) with ‘finger-type’ endings and η-type levels II–III margin undulation patterns. (**f**) interdigitating multicellular structures from sample 59 with shorter ‘wave-type’ endings and η-type levels II–III margin undulation patterns. Scale bars are 50 µm.

**Figure 6:**
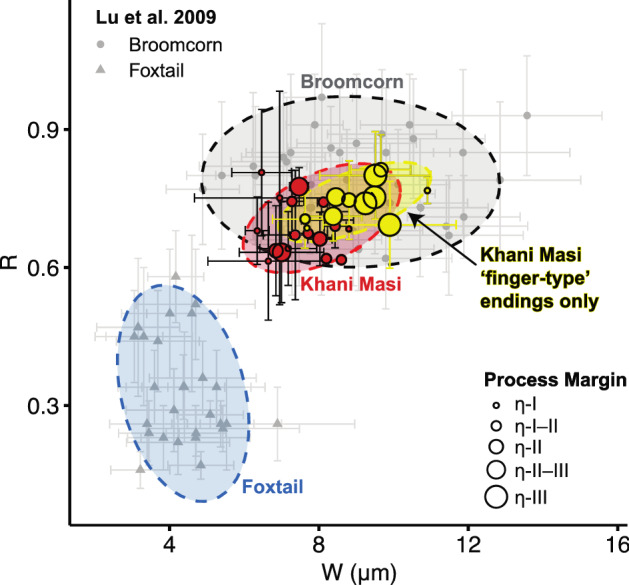
A plot of average morphometric values, R and W, for each Khani Masi interdigitating multicellular structure (red and yellow) compared to values reported by Lu et al.^77^ (light gray circles and triangles). W (x-axis) is the long cell (elongate) ending length (Table S6, criteria 4), and R (y-axis) is the ratio of processes amplitude to endings (Table S6, criteria 5). The distribution of Khani Masi multicells with exclusively ‘finger-type’ long cell endings (yellow) falls completely with the normal distribution ellipse for broomcorm millet as reported by Lu et al.^77^ (dark gray). All points represent average values with standard deviation error bars. Khani Masi (red and yellow) point size indicates the level (I–III) of the η-type undulation pattern. Normal distribution ellipses are colored by broomcorn millet (dark gray), foxtail millet (blue), all Khani Masi multicells (red), and only Khani Masi multicells with ‘finger-type’ long cell endings (yellow). Figure modified from Lu et al.^77^.

This study identified 30 phytolith multicellular structures (silica skeletons), with characteristics consistent with the inflorescence bracts of broomcorn millet (*Panicum miliaceum*) (Figs. 5, 6 and Supplementary Table S7). Multicellular structures sizes range between 1 and 10 individual measurable interdigitating phytolith morphotypes (average size: 3) for a total of 90 individually measured phytoliths. Note that partial or broken morphotypes at the edges of the multicellular structures were not measured. 28 of the 30 interdigitating multicellular structures meet five criteria and 19 meet all six criteria (Supplementary Table S7). In every interdigitating multicellular structure, papillate phytoliths are absent (*criteria 1,* Supplementary Table S5), margin processes are η-type (*criteria 2*), and the process height to body ratio is greater than 1 (*criteria 6*). 13 multicellular structures have distinctly finger-type endings, and 14 have either both ending types or intermediate appearances (*criteria 3*). The overall averages for both long cell (elongate) ending lengths (W = 8.13 ± 1.10 µm, *criteria 4*) and the ratio of process height to ending length (R = 0.71 ± 0.06 µm, *criteria 5*) are within one standard deviation of the values reported by Lu et al.^77^ for *P. miliaceum*. All measurements of both the individual and multicellular interdigitating phytoliths exceed the minimum required sample size for ensuring means are within 5% of actual population means at a 90% confidence level (Supplementary Table S8)^87,88^.

Of the species native to Iraq with known interdigitating inflorescence phytoliths, only *Paniucm repens* (torpedo grass) shares four of the same diagnostic criteria with *Panicum miliaceum*. *P. repens* only produces short, ‘wavy-type’ endings, whereas *P. miliaceum* mostly produces ‘finger-type’ endings (*criteria 3*)^77,78^. Nineteen interdigitating multicellular structures have finger or both finger- and wavy-type endings making them potentially distinct from *P. repens*. However, cautiously using the stricter criteria of exclusively ‘finger-type’ endings, eleven multicellular structures are securely identified as *P. miliaceum* (highlighted yellow in Fig. 6). Because several species can produce η-type level I–II margin processes, an even more conservative identification of *P. miliaceum* could also require η-type level III margin processes. In this case, six interdigitating multicellular structures have both η-type level II–III or III margin processes and exclusively ‘finger-type’ endings, increasing confidence for the identification of *P. miliaceum* at Khani Masi.

It is possible that the interdigitating phytolith morphotypes analyzed in this study could potentially be produced from some yet unknown wild C_4_ plant that has phytoliths identical to those of *Panicum miliaceum*. However, based on the current phytolith knowledge, the interdigitating phytolith morphotypes measures fit remarkably well within the broomcorn population. For example, *Panicum turgidum* Forssk. (desert grass) is not well studied from a phytolith perspective, although it does not appear to resemble the strict criteria for *P. miliaceum*^89,90^. *Panicum turgidum* is also adapted to and moderately present only in the sandy desert regions in the extreme southeast of Iraq (Saharo-Sindian), and is not expected to grow in the moister Khani Masi region^17,91^. Future research should focus on generating accessible phytolith references for the wild grasses of Iraq and the greater Mesopotamian region.

## Discussion

Our results demonstrate that broomcorn millet (*Panicum miliaceum*) is present at the mid-second millennium BCE site Khani Masi located along the Upper Diyala/Sirwan River in northern Iraq. This result represents the first phytolith identification of *P. miliaceum* from ancient Iraq and aligns with both contemporary textual sources and some regional macrobotanical evidence that suggests millet was present in Mesopotamia at this time.

The presence of millet at Khani Masi may also provide the earliest evidence to-date for regional cereal multi-cropping in Mesopotamia. That is, in addition to the winter cereals, wheat and barley, which are attested in both the micro- and macro-botanical records at the site^51,54^, we also now have robust micro-botanical evidence for summer grains. Although the presence of a plant is not enough to prove it was cultivated^92^, the discovery of a non-native, east Asian domesticate outside its environmental niche and within a dung-rich context strongly suggests that it may have been cultivated as a forage crop. If cultivated locally at Khani Masi, millet-as-forage provides a “long prelude”^93^ or “clear antecedent”^41^ for its use in the region’s first millennium BCE agricultural intensification systems. Locally cultivated millet also suggests that previous arguments for environmental constraints on early millet cultivation may be overstated and require alternative explanations.

While Mesopotamia is (semi-)arid in terms of precipitation, it contains an abundance of perennial water sources that could support summer cereal cultivation without major investments in irrigation. As further articulated below, our results suggest that the absence of evidence for millet in previous investigations is due to the particularly strong taphonomic bias against millet grain preservation as well as to the low archaeological visibility of pastoral lifeways. Thus, millet cultivation was likely far more widespread in the second millennium BCE than is currently recognized. Furthermore, the availability of millet as an alternative food source for people or animals requires the reassessment of Bronze Age models of urban provisioning, resilience, and human-environment interactions.

### Summer cultivation in (semi-)arid Mesopotamia

Mesopotamia (ancient Iraq) has a long history of irrigation and a myriad of perennial water sources, particularly in the ecologically diverse Zagros foothills zone, and the cultivation of millet in the region should be unsurprising. Irrigation technology was developed in the region during the sixth–fifth millennium BCE^94,95^ meaning it had been practiced for millennia by the second millennium BCE. That sesame is cultivated in the region from the third millennium BCE onwards further emphasizes that Mesopotamian communities were familiar with summer crops and were versed in small-scale summer cultivation. Thus, while the local seasonal climate may be unfavorable for precipitation-based summer cultivation, river discharge rates during April–June suggest that preexisting irrigation infrastructure could easily have been used to irrigate millet sown as a cover crop in river flood control areas or in fallow fields^16,60,96^ (see Fig. 2c). Summer crops also could be grown opportunistically outside winter cultivation areas along perennial water sources such as riverbanks and karstic springs.

Two alternative scenarios, although unlikely, could explain the consumption of millet by animals at Khani Masi without local cultivation: (a) the feral growth of the domesticated grain near perennial water sources or (b) the long-distance transport of unhulled grains from their natural growing range.

First, the feral growth of domesticated *Panicum miliaceum* around nearby perennial water sources is unlikely but cannot be completely ruled out. Feral growth requires that broomcorn millet was introduced into the region before the midden was formed at Khani Masi, during late third–early second millennium BCE. If this was the case, the implication is that domesticated Broomcorn millet was still known and actively used for animal forage at this time, centuries earlier than previously accepted.

Second, Khani Masi is located along a strategic trade route connecting lowland Mesopotamia to the Iranian Zagros highlands and Central Asia^51,52^. Yet, it is unlikely that sufficient amounts of unhulled millet grains would have been transported hundreds of kilometers from the Taurus-Zagros mountains or from maritime ports in lowland Mesopotamia only to be used as fodder for local sheep-goats (Supplementary Fig. S3). Moreover, the maximum estimated one-way travel distance for sheep and goat herds based on average consumption to defecation times is 35–47 kilometers^97^ and does not allow for a scenario in which millet was consumed in its natural range and deposited at Khani Masi (Supplementary Fig. S3). Thus, the most parsimonious explanation is that a small number of seeds were transported and planted locally. Furthermore, the presence of *Panicum miliaceum* in multiple layers in Trench Y82 suggests it was cultivated in small quantities over multiple years (Table S1).

### Factors affecting broomcorn millet preservation and recovery

By the mid-second millennium BCE, Mesopotamia is demonstrably integrated into the globalized networks that connected all of Eurasia^98–100^. The technology and ecological niches required for summer cultivation were present, and both millet and sesame are mentioned concurrently in textual sources. Macrobotanical evidence of millet has been scarce across Mesopotamia, but now millet micro-remains are verified at Khani Masi. Together, these lines of evidence suggest that a combination of taphonomic and cultural factors are affecting the regional recovery of millet.

#### Taphonomic factors

As with most archaeological material, several factors condition both the entrance of plant remains into settlement areas and their preservation after arrival^101^. In other ethnographic and archaeological case studies, the lack of macrobotanical evidence for millet cultivation has been attributed to its minor cultivation, processing in off-site areas, and particular susceptibility to destructive taphonomic processes^92,102^. While millet may have been a minor crop in second millennium BCE Mesopotamia, its paucity in the long-term archaeobotanical record is most likely the result of the fragility of millet grains coupled with regionally poor macrobotanical preservation.

Compared to other crops, millet’s small inflorescence structures and high fat, oil-rich grains make it particularly susceptible to destruction during charring^5,92,103^––the primary mechanism for chaff and grain preservation in most regions^104,105^. Accordingly, multi-proxy methods are required to investigate millet processing even in regions where millet is a major cereal crop^102,103^. Notably, in regions where millet was introduced and not anticipated, highly degraded charred grains may be mistaken for weeds^44^.

In Southwest Asia, preservation conditions for macrobotanicals can be poor, especially in shallower sites, and the ubiquities of even the major crops, wheat and barley, can sometimes be too low for meaningful statistical analysis (e.g.^106^). For millet, macrobotanical finds are remarkably rare in all periods, even in periods when millet is intensively cultivated^38^. Millet finds from Southwest Asia seem to be restricted to a single grain for an entire site (e.g.^46,49,107^) or large caches recovered under exceptional preservation conditions such as roof storage collapse from catastrophic fires (Tille Höyük, Turkey and Haftavan, Iran^32^); or in jars with tar (Nimrud^39^).

In many ways, the archaeobotanical record for millet mirrors that of sesame (*Sesamum indicum* L.), another small-grained, oil-rich summer plant translocated into Mesopotamia. Like millet, sesame seeds are also textually attested but exceedingly rare in the archaeobotanical record^29^. Their small size and high oil content make carbonized sesame seeds extremely fragile and prone to disintegrate during the recovery process. Further, their relatively small quantities and processing in off-site areas make them less likely to enter the archaeological record in the first place^29,108^. Consequently, no sesame grains are attested in Mesopotamia for the nearly 1000 years between the earliest grains recovered ca. 2300 BCE (Tell Abu Salabikh, Iraq) and those dating to the late second millennium BCE^6,33,109^. Where data are published, sesame finds, too, are restricted to very few grains or large caches^29^. However, like millet, recent proteomic, residue, and microbotanical approaches are demonstrating that sesame and other exotic plants were more widespread in second millennium BCE Southwest Asia than previously thought^100,110,111^.

#### Cultural factors

The context in which millet was recovered at Khani Masi suggests an additional taphonomic reason why millet is rare in the second millennium BCE: its primary use as animal forage (or fodder). In contrast to sesame, millet phytoliths at Khani Masi were primarily recovered from burned and discarded dung-rich sediment that suggests introduction via animal dung^54^. Archaeologists and biologists alike have long appreciated the facts hidden in animal waste^112,113^, but the value of dung and its contents is still underappreciated for investigating Mesopotamia’s economies and ecologies.

Dung-associated plant material is subjected to additional destructive processes that decrease the likelihood of grain identification from macrobotanicals. First, dung is most likely to enter the archaeological record through fuel use and animal penning (although evidence is still pending for dung as a common construction material in Mesopotamia). Second, unlike wild seeds, which are abundant in ruminant animal dung, domesticated cereal grains are starchy or oil-rich with thin protective outer coatings and rarely survive sheep and goat digestion^114–116^. Third, because dung in ancient settlements is often used as fuel or burned to reduce the volume of dung accumulating in animal penning areas, any fragile millet grains that survive digestion would be subsequently destroyed through burning. Finally, discarded organic rich dung and ashes often decay after deposition (diagenesis) further destroying organic macrobotanical evidence^114^.

The strong taphonomic bias against millet grain preservation means that this grain has been below our ability to resolve using traditional macrobotanical methods^77^. Microbotanical and geochemical approaches (e.g., phytoliths, dung spherulites, FTIR), which can effectively identify animal dung and its contents, have not yet been widely used in the region. This study demonstrates, however, that millet was cultivated in Mesopotamia and that phytolith analyses of dung deposits are likely key for investigating the role of forage and fodder in the advent of regional multi-cropping.

### The pastoral origins of multi-cropping in Mesopotamia

The recovery of millet from animal dung—consumed as a forage crop—suggests that the initial practice of multi-cropping in Mesopotamia is likely associated with small-scale pastoral diversification strategies—not imperial agricultural mandates. Pastoral, here, is defined broadly as the husbandry of sheep-goats (after^117,118^), acknowledging that local pastoral systems and their specific practices, level of mobility, and integration into agricultural systems vary widely. Millet grown as a forage crop would be directly consumed by animals, not harvested. The pastoral origins of multi-cropping in Mesopotamia complement multiple botanical and isotopic studies from across Central and South Asia that also suggest millet was adopted slowly, through bottom-up, pastoral initiatives^13,22,41,93,119–123^. Millet’s low investment, high return qualities made it especially well-suited to the needs of the semi-mobile pastoralists who transported it across Central Asia’s ecologically diverse landscapes^5,124^. It is fitting then that this new crop may have been first adopted by pastoralists living in the environmentally complex Mesopotamian-Zagros interface.

Pastoral practice outside of institutional spheres has been a topic of intense debate in Mesopotamian archaeology because it is not well documented in Mesopotamia’s archaeological or textual records^125^. However, we should consider that the introduction of new foods and related practices likely disrupted lifeways^18^. As well as enhancing agro-pastoral resilience through diversification, millet may have been a destabilizing force by offering increased autonomy from established (or distant) socio-political and economic systems^126,127^. In both cases, the possible pastoral origins of multi-cropping highlight the influence of steppe region pastoral practice on the political and land use histories of Southwest Asia. Many Southwest Asian crops and animals were first domesticated in the Zagros foothills (“hilly flanks”)^128,129^, and this study suggests that the Zagros foothills may have continued to be a regional center of agro-pastoral innovation for Mesopotamia during the Bronze Age.

### Reassessing provisioning models in light of food globalization

Beyond the Zagros Region, the adoption of millet likely had far-reaching impacts on Mesopotamia’s social, political, and economic systems beginning in the second millennium BCE. The verified presence of millet in mid-second millennium BCE Mesopotamia sheds new light on historical events and trajectories of the region and requires a reassessment of models of urban provisioning, resilience, and human–environment interactions. For example, Lawrence et al.^36^ attribute the “decoupling” of urban site size (and population) with climate trends after 2000 BCE and urban size with sustaining area after 1200 BCE to changes in labor organization, taxation, and integration into long-distance trade networks. However, like most models of Mesopotamian economies, they have not yet explicitly considered the impact of new crops^36,37^. However, this decoupling of demographic and environmental variables coincides with the arrival of new crops with properties optimally suited to diversifying and strengthening the resilience of Mesopotamia’s agro-pastoral production systems. Even a low-level or opportunistic cultivation of millet, for human or animal consumption, may have had a significant impact on urban provisioning and thus resilience capacity^130^. Future studies could further investigate the origins of multi-cropping by investigating isotopic δ^13^C enrichment from low-level millet (C_4_) consumption and by deploying microscopic methods that acknowledge the taphonomic biases against millet grain preservation. Perhaps uncoincidentally, evidence of millet cultivation is nearly as rare as studies using isotopic^131^ and phytolith approaches^54^ with potentially critical impacts on our understanding of Mesopotamia’s social, political, and economic systems.

## Conclusion

Here we provide the earliest microbotanical evidence of broomcorn millet (*Panicum miliaceum*) in Mesopotamia (ancient Iraq) and suggest that the origins of multi-cropping (summer cultivation) begin in the second millennium BCE. This finding aligns with ongoing investigations of early food globalization across Eurasia, a conversation in which Mesopotamia has been notably absent. As in other regions, the initial use of millet in Mesopotamia was likely as a foraging crop. Agro-pastoralists in the Zagros-Mesopotamian interface may have grown millet opportunistically at low levels for centuries as a diversification strategy^41,132^ before it was considered food suitable for human consumption or economically advantageous^12,93^ to the political economies within the first millennium BCE Neo-Assyrian Empire. Strong taphonomic bias against millet grain preservation provides an explanation for why its recovery has been so rare despite its known presence in textual sources. Micro-remain analysis offers a promising path forward for exploring the processes and practice of multi-cropping in Mesopotamia. In fact, this study highlights that micro-remain analyses have the potential to fundamentally transform our understanding of daily life, the formation of states and empires, and human-environment relationships in one of the most prominent and strategic nodes of ancient Eurasian and African networks.

## Materials and methods

### Excavation and sampling

Two adjacent trenches (10 × 2.5m^2^) separated by a 0.5 m baulk were excavated in area Y82 at Khani Masi (SRP46) by the Sirwan Regional Project (SRP). Charcoal samples for ^14^C dating were collected during excavation and analyzed at the University of Arizona AMS Laboratory. ^14^C date ranges were calibrated using OxCal v4.4.4 (Fig. 4 and Supplementary Tables S3–4)^65^. Bulk sediment samples (~ 30 g) were collected in plastic bags directly from the freshly cleaned baulk section, and sampling tools were cleaned with acetone between every sample. Sample locations were tagged, photographed, and geolocated using an Emlid RS + RTK GNSS system.

### Microscopy

Phytoliths were extracted using the Katz et al.^133^ method. Phytoliths were identified and photographed using a Nikon eclipse LV100N POL petrographic microscope at 200× and 400× magnification. Morphological identification followed the standard literature^66,134–137^ using the International Code for Phytolith Nomenclature (ICPN 2.0) when possible^67^.

### Morphometric analysis

Quantitative phytolith measurements were taken in ImageJ software (version 1.5.3) using the morphometric criteria defined by^76,77,82^ (Supplementary Fig. S2 and Table S6). To avoid any taphonomical bias in the morphometric analysis, we measured only complete individual phytoliths forming multicellular structures (silica skeletons). Partial or broken individual phytoliths at the edges of each silica skeleton were not measured. Following Ball et al.^87,88^, minimum sample sizes were calculated for all measurements for both multicellular structures and individual phytoliths to ensure sample means were within 5% of the actual population means at a 90% confidence level (Supplementary Table S8).

## Supplementary Information

Supplementary Information.

## References

[1] Andrews DJ, Kassam AH, Papendick RI, Sanchez PA, Triplett GB (1976). The importance of multiple cropping in increasing world food supplies. Multiple Cropping.

[2] Gallaher RN, Hudson RJ (2009). Multiple cropping systems. Management of Agricultural, Forestry, and Fisheries Enterprises.

[3] Petrie CA, Bates J (2017). ‘Multi-cropping’, intercropping and adaptation to variable environments in indus south asia. J. World Prehistory.

[4] Spengler RN (2019). Fruit From the Sands: The Silk Road Origins of the Foods We Eat.

[5] Miller NF, Spengler RN, Frachetti M (2016). Millet cultivation across Eurasia: Origins, spread, and the influence of seasonal climate. The Holocene.

[6] Zohary D, Hopf M, Weiss E (2012). Domestication of Plants in the Old World: The Origin and Spread of Cultivated Plants in West Asia, Europe and the Nile Valley.

[7] Amadou I, Gounga ME, Le G-W (2013). Millets: Nutritional composition, some health benefits and processing: A review. Emir. J. Food Agric..

[8] Lyon, D. J. *et al.* Producing and Marketing Proso Millet in the Great Plains. *Univ. Neb. Ext. Circ.* **#EC137** (2008).

[9] Rachie KO (1975). The Millets: Importance, Utilization and Outlook.

[10] Fuller DQ, Boivin N, Hoogervorst T, Allaby R (2011). Across the Indian Ocean: The prehistoric movement of plants and animals. Antiquity.

[11] Jones M (2016). Food globalisation in prehistory: The agrarian foundations of an interconnected continent. J. Br. Acad..

[12] Jones M (2011). Food globalization in prehistory. World Archaeol..

[13] Liu X (2019). From ecological opportunism to multi-cropping: Mapping food globalisation in prehistory. Quat. Sci. Rev..

[14] Sherratt A, Mair VH (2006). The Trans-Eurasian exchange: The prehistory of Chinese relations with the West. Contact and Exchange in the Ancient World.

[15] Wirth E (1962). Agrargeographie des Irak.

[16] FAO/GIEWS. *FAO GIEWS Country Brief on Iraq*. http://www.fao.org/giews/countrybrief/country.jsp?lang=en&code=IRQ (2020).

[17] Bor NL, Townsend CC, Guest E, Al-Rawi A (1968). Gramineae. Flora of Iraq, Gramineae.

[18] Rosenzweig MS, MacGinnis J, Wicke D, Greenfield T (2016). ‘Ordering the chaotic periphery’: The environmental impact of the neo-assyrian empire on its provinces. The Provincial Archaeology of the Assyrian Empire.

[19] Fuller DQ, Boivin N (2009). Crops, cattle and commensals across the Indian Ocean: Current and Potential Archaeobiological Evidence. Études Océan Indien.

[20] Barjamovic G, Kristiansen K, Lindkvist T, Myrdal J (2018). Interlocking commercial networks and the infrastructure of trade in western asia during the bronze age. Trade and Civilisation: Economic Networks and Cultural Ties from Prehistory to the Early Modern Era.

[21] Frachetti MD, Smith CE, Traub CM, Williams T (2017). Nomadic ecology shaped the highland geography of Asia’s Silk Roads. Nature.

[22] Spengler R (2014). Early agriculture and crop transmission among Bronze Age mobile pastoralists of Central Eurasia. Proc. R. Soc. B Biol. Sci..

[23] Fick SE, Hijmans RJ (2017). WorldClim 2: New 1-km spatial resolution climate surfaces for global land areas. Int. J. Climatol..

[24] Williams T (2014). The Silk Roads: An ICOMOS Thematic Study.

[25] Charles M, Postgate JN, Powell MA (1984). Introductory remarks on the cereals. Bulletin on Sumerian Agriculture.

[26] Powell MA, Postgate JN, Powell MA (1984). Sumerian cereal crops. Bulletin on Sumerian Agriculture.

[27] *The Assyrian Dictionary of the Oriental Institute of the University of Chicago: D*. (ed. Oppenheim A.L. et al.) vol. 3 (Oriental Institute of the University of Chicago, 1959).

[28] Widell M, Wilkinson TJ, Gibson M, Widell M (2013). Staple production, cultivation and sedentary life: Model Input data. Models of Mesopotamian Landscapes: How Small-Scale Processes Contributed to the Growth of Early Civilizations.

[29] Bedigian D, Postgate JN, Powell MA (1985). Is še-giš-ì Sesame or Flax?. Bulletin on Sumerian Agriculture.

[30] Waetzoldt H, Postgate JN, Powell MA (1985). Ölpflanzen und Pflanzenöle im 3. Jahrtausend. Bulletin on Sumerian Agriculture.

[31] Maekawa K, Postgate JN, Powell MA (1984). Cereal Cultivation in the Ur III period. Bulletin on Sumerian Agriculture.

[32] Nesbitt M, Summers GD (1988). Some recent Discoveries of Millet (*Panicum Miliaceum* L. and *Setaria italica* (L.) P. Beauv.) at Excavations in Turkey and Iran. Anatol. Stud..

[33] Charles M, Green A (1993). Botanical remains. Abu Salabikh Excavations: The 6G Ash-Tip and Its Contents: Cultic and Administrative Discard from the Temple?.

[34] Postgate JN (1992). Early Mesopotamia: Society and Economy at the Dawn of History.

[35] *Bulletin on Sumerian Agriculture*. (eds. Postgate, J. N. & Powell, M. A.) vol. 1 (University of Cambridge, 1984).

[36] Lawrence D, Philip G, Hunt H, Snape-Kennedy L, Wilkinson TJ (2016). Long term population, city size and climate trends in the fertile crescent: A first approximation. PLoS ONE.

[37] *Models of Mesopotamian landscapes: How small-scale processes contributed to the growth of early civilizations.* (eds. Wilkinson, T.J., Gibson, M., & Widell, M.) (Archaeopress, 2013).

[38] Charles, M. & Dobney, K. *Mesopotamian Environmental Archaeology Database: Phase I Iraq *. (Archaeology Data Service [distributor], 2009).

[39] Helbaek H, Mallowan MEL (1966). The Plant Remains from Nimrud. Nimrud and its Remains.

[40] Boserup E (1965). The Conditions of Agricultural Growth: The Economics of Agrarian Change Under Population Pressure.

[41] Brite EB, Kidd FJ, Betts A, Negus Cleary M (2017). Millet cultivation in Central Asia: A response to Miller et al.. The Holocene.

[42] Helbaek H, Braidwood RJ, Howe B (1960). The Paleoethnobotany of the Near East and Europe. Prehistoric Investigations in Iraqi Kurdistan.

[43] Jacobsen T (1982). Salinity and Irrigation Agriculture in Antiquity: Diyala Basin Archaeological Projects: Report on Essential Results, 1957–58.

[44] Motuzaite-Matuzeviciute G, Staff RA, Hunt HV, Liu X, Jones MK (2013). The early chronology of broomcorn millet (Panicum miliaceum) in Europe. Antiquity.

[45] Field H (1932). Ancient Wheat and Barley from Kish, Mesopotamia. Am. Anthropol..

[46] Wengrow D (2016). Gurga Chiya and Tepe Marani: New Excavations in the Shahrizor Plain, Iraqi Kurdistan. Iraq.

[47] van Zeist W, Kühne H (2008). Comments on Plant Cultivation at Two Sites on the Khabur, North-Eastern Syria. Umwelt und Subsistenze der assyrischen Stadt Dur-Katlimmu am unteren Habur.

[48] van Zeist, W. Comments on plant cultivation at two sites on the Khabur, North-eastern Syria. in *Reports on archaeobotanical studies in the Old World.* (ed. van Zeist, W.) 33–60 (2003).

[49] Riehl S (2000). Erste ergebnisse der archäobotanischen untersuchungen in der zentralen oberstadt von Tall Mozan/Urkeš im rahmen der DOG-IIMAS-Kooperation. Mitteilungen Dtsch. Orient-Ges. Zu Berl..

[50] Riehl S, Deckers K, Doll M, Pfälzner P, Riehl S (2010). Plant production in a changing environment: The archaeobotanical remains from Tell Mozan. Development of the Environment, Subsistence and Settlement of the City of Urkeš and Its Region.

[51] Glatz C (2019). Babylonian Encounters in the Upper Diyala Valley: Contextualizing the Results of Regional Survey and the 2016–2017 Excavations at Khani Masi. Am. J. Archaeol..

[52] Glatz C, Casana J (2016). Of highland-lowland borderlands: Local societies and foreign power in the zagros-mesopotamian interface. J. Anthropol. Archaeol..

[53] Perruchini E, Glatz C, Hald MM, Casana J, Toney JL (2018). Revealing invisible brews: A new approach to the chemical identification of ancient beer. J. Archaeol. Sci..

[54] Laugier EJ, Casana J, Glatz C, Sameen SM, Cabanes D (2021). Reconstructing agro-pastoral practice in the Mesopotamian-Zagros borderlands: Insights from phytolith and FTIR analysis of a dung-rich deposit. J. Archaeol. Sci. Rep..

[55] Zohary M (1973). Geobotanical foundations of the Middle East.

[56] Sinha A (2019). Role of climate in the rise and fall of the Neo-Assyrian Empire. Sci. Adv..

[57] Schneider, U., Becker, A., Finger, P., Rustemeier, E. & Ziese, M. GPCC Full Data Monthly Product Version 2020 at 0.25°: Monthly Land-Surface Precipitation from Rain-Gauges built on GTS-based and Historical Data. (2020).

[58] Casana J, Glatz C (2017). The land behind the land behind baghdad: Archaeological landscapes of the upper Diyala (Sirwan) River Valley. Iraq.

[59] Wilkinson TJ, Jas RM (2000). Settlement and land use in the zone of uncertainty in upper mesopotamia. Rainfall and Agriculture in Northern Mesopotamia.

[60] Saleh, D. K. *Stream gage descriptions and streamflow statistics for sites in the Tigris River and Euphrates River basins, Iraq*. (US Department of the Interior, US Geological Survey Reston, VA, USA, 2010).

[61] Bar-Oz G (2019). Ancient trash mounds unravel urban collapse a century before the end of Byzantine hegemony in the southern Levant. Proc. Natl. Acad. Sci..

[62] Shillito L-M, Matthews W (2013). Geoarchaeological investigations of midden-formation processes in the early to late ceramic neolithic levels at Çatalhöyük, Turkey ca. 8550–8370 cal BP. Geoarchaeology.

[63] McCorriston J, Weisberg S (2002). Spatial and temporal variation in mesopotamian agricultural practices in the Khabur Basin, Syrian Jazira. J. Archaeol. Sci..

[64] Stone EC (1987). Nippur neighborhoods.

[65] Bronk Ramsey, C. *OxCal v4.4.4*. (2021).

[66] Madella M, Alexandre A, Ball T (2005). International code for phytolith nomenclature 1.0. Ann. Bot..

[67] Neumann K (2019). International code for phytolith nomenclature (ICPN) 2.0. Ann. Bot..

[68] Ge Y, Lu H, Zhang J, Wang C, Gao X (2020). Phytoliths in inflorescence bracts: Preliminary results of an investigation on common panicoideae plants in China. Front. Plant Sci..

[69] Parry DW, Hodson MJ (1982). Silica distribution in the caryopsis and inflorescence bracts of foxtail millet [Setaria italica (L.) Beauv.] and its Possible Significance in Carcinogenesis. Ann. Bot..

[70] Cabanes D (2012). Human impact around settlement sites: A phytolith and mineralogical study for assessing site boundaries, phytolith preservation, and implications for spatial reconstructions using plant remains. J. Archaeol. Sci..

[71] Cabanes D, Shahack-Gross R (2015). Understanding fossil phytolith preservation: The role of partial dissolution in paleoecology and archaeology. PLoS ONE.

[72] Li Z, de Tombeur F, Linden CV, Cornelis J-T, Delvaux B (2020). Soil microaggregates store phytoliths in a sandy loam. Geoderma.

[73] Goldberg P, Macphail RI (2006). Practical and Theoretical Geoarchaeology.

[74] Ball TB (2016). Phytoliths as a tool for investigations of agricultural origins and dispersals around the world. J. Archaeol. Sci..

[75] Kealhofer L, Huang F, DeVincenzi M, Kim MM (2015). Phytoliths in Chinese foxtail millet (*Setaria italica*). Rev. Palaeobot. Palynol..

[76] Weisskopf AR, Lee G-A (2016). Phytolith identification criteria for foxtail and broomcorn millets: A new approach to calculating crop ratios. Archaeol. Anthropol. Sci..

[77] Lu H (2009). Phytoliths analysis for the discrimination of foxtail millet (*Setaria italica*) and common millet (*Panicum miliaceum*). PLoS ONE.

[78] Zhang J (2018). Phytolith analysis for differentiating between broomcorn millet (*Panicum miliaceum*) and its weed/feral type (*Panicum ruderale*). Sci. Rep..

[79] Nesbitt M (2006). Identification Guide for Near Eastern Grass Seeds.

[80] Rudov A, Mashkour M, Djamali M, Akhani H (2020). A review of C4 plants in southwest asia: An ecological, geographical and taxonomical analysis of a region with high diversity of C4 eudicots. Front. Plant Sci..

[81] Weber SA, Fuller DQ (2008). Millets and their role in early agriculture. Pragdhara.

[82] Zhang J, Lu H, Wu N, Yang X, Diao X (2011). Phytolith analysis for differentiating between foxtail millet (*Setaria italica*) and green foxtail (*Setaria viridis*). PLoS ONE.

[83] Out WA, Madella M (2016). Morphometric distinction between bilobate phytoliths from *Panicum miliaceum* and *Setaria italica* leaves. Archaeol. Anthropol. Sci..

[84] Bhat MA, Shakoor SA, Badgal P, Soodan AS (2018). *Taxonomic Demarcation of Setaria pumila (Poir.) Roem. & Schult., S. verticillata (L.) P. Beauv., and S. viridis (L.) P. Beauv. (Cenchrinae, Paniceae, Panicoideae, Poaceae) From Phytolith Signatures*. Front. Plant Sci..

[85] Ge Y (2018). Phytolith analysis for the identification of barnyard millet (*Echinochloa* sp.) and its implications. Archaeol. Anthropol. Sci..

[86] Madella M, Lancelotti C, García-Granero JJ (2016). Millet microremains—an alternative approach to understand cultivation and use of critical crops in Prehistory. Archaeol. Anthropol. Sci..

[87] Ball TB, Vrydaghs L, Van Den Hauwe I, Manwaring J, De Langhe E (2006). Differentiating banana phytoliths: Wild and edible *Musa acuminata* and *Musa balbisiana*. J. Archaeol. Sci..

[88] Ball TB (2016). Morphometric analysis of phytoliths: Recommendations towards standardization from the International Committee for Phytolith Morphometrics. J. Archaeol. Sci..

[89] Hunt HV (2008). Millets across Eurasia: Chronology and context of early records of the genera *Panicum* and *Setaria* from archaeological sites in the Old World. Veg. Hist. Archaeobotany.

[90] Weisskopf AR (2014). Millets, Rice and Farmers: Phytoliths as Indicators of Agricultural, Social and Ecological Change in Neolithic and Bronze Age Central China.

[91] Ghazanfar SA, McDaniel T (2016). Floras of the Middle East: A Quantitative Analysis and Biogeography of the Flora of Iraq. Edinb. J. Bot..

[92] Reddy SN (1997). If the threshing floor could talk: Integration of agriculture and pastoralism during the late harappan in Gujarat, India. J. Anthropol. Archaeol..

[93] Liu X, Jones MK (2014). Food globalisation in prehistory: Top down or bottom up?. Antiquity.

[94] Adams RMcC (1981). Heartland of Cities: Surveys of Ancient Settlement and Land Use on the Central Floodplain of the Euphrates.

[95] Oates J (1969). Choga Mami, 1967–68: A Preliminary Report. Iraq.

[96] Rost S (2019). Navigating the ancient Tigris: Insights into water management in an early state. J. Anthropol. Archaeol..

[97] Dunseth ZC (2019). Archaeobotanical proxies and archaeological interpretation: A comparative study of phytoliths, pollen and seeds in dung pellets and refuse deposits at Early Islamic Shivta, Negev, Israel. Quat. Sci. Rev..

[98] *Amarna Diplomacy: The Beginnings of International Relations*. (Johns Hopkins Univeristy Press, 2000).

[99] Kenoyer JM, Olijdam E, Spoor RH (2008). Indus and Mesopotamian Trade Networks: New Insights from Shell and Carnelian Artifacts. Intercultural Relations Between South and Southwest Asia. Studies In Commemoration Of E.C.L.During Caspers (1934–1996).

[100] Scott A (2020). Exotic foods reveal contact between South Asia and the Near East during the second millennium BCE. Proc. Natl. Acad. Sci..

[101] Schiffer MB (1987). Formation Processes of the Archaeological Record.

[102] Bates J, Singh RN, Petrie CA (2017). Exploring Indus crop processing: Combining phytolith and macrobotanical analyses to consider the organisation of agriculture in northwest India c. 3200–1500 bc. Veg. Hist. Archaeobotany.

[103] Harvey EL, Fuller DQ (2005). Investigating crop processing using phytolith analysis: The example of rice and millets. J. Archaeol. Sci..

[104] Hillman, G. Interpretation of archaeological plant remains: The application of ethnographic models from Turkey. in *Plants and Ancient Man: Studies in Palaeoethnobotany: Proceedings of the Sixth Symposium of the International Work Group for Palaeoethnobotany, Groningen, 30 May-3 June 1983* (eds. van Zeist, W. & Casparie, W. A.) 1–41 (Balkema, 1984).

[105] Hillman G, Mercer R (1981). Reconstructing crop husbandry practices from charred remains of crops. Farming Practice in British prehistory.

[106] Helbaek H (1972). Samarran irrigation agriculture at Choga Mami in Iraq. Iraq.

[107] Miller NF (1982). Economy and Environment of Malyan, a Third Millennium BC Urban Center in Southern Iran.

[108] Bedigian D (2010). Sesame: The Genus Sesamum.

[109] Van Zeist W, Gasche H, Tanret M (1994). Some notes on second millennium BC plant cultivation in the Syrian Jazira. Cinquante-deux réflexions sur le Proche-Orient ancien offertes en hommage a Leon de Meijer.

[110] Linares V (2019). First evidence for vanillin in the old world: Its use as mortuary offering in Middle Bronze Canaan. J. Archaeol. Sci. Rep..

[111] Chowdhury MP, Campbell S, Buckley M (2021). Proteomic analysis of archaeological ceramics from Tell Khaiber, southern Iraq. J. Archaeol. Sci..

[112] Miller N, Smart T (1984). Intentional burning of dung as fuel: A mechanism for the incorporation of charred seeds into the archaeological record. J. Ethnobiol..

[113] Putman RJ (1984). Facts from faeces. Mammal Rev..

[114] Shahack-Gross R (2011). Herbivorous livestock dung: Formation, taphonomy, methods for identification, and archaeological significance. J. Archaeol. Sci..

[115] Valamoti SM, Charles M (2005). Distinguishing food from fodder through the study of charred plant remains: An experimental approach to dung-derived chaff. Veg. Hist. Archaeobotany.

[116] Wallace M, Charles M (2013). What goes in does not always come out: The impact of the ruminant digestive system of sheep on plant material, and its importance for the interpretation of dung-derived archaeobotanical assemblages. Environ. Archaeol..

[117] Hammer EL, Arbuckle BS (2017). 10,000 Years of pastoralism in anatolia: A review of evidence for variability in pastoral lifeways. Nomadic Peoples.

[118] Meadow RH, Bar-Yosef O, Khazanov AM (1992). Inconclusive remarks on pastoralism, nomadism, and other animal-related matters. Pastoralism in the Levant: Archaeological Materials in Anthropological Perspectives.

[119] Frachetti MD (2012). Multiregional emergence of mobile pastoralism and nonuniform institutional complexity across Eurasia. Curr. Anthropol..

[120] García-Granero JJ, Lancelotti C, Madella M, Ajithprasad P (2016). Millets and herders: The origins of plant cultivation in semiarid North Gujarat (India). Curr. Anthropol..

[121] Hermes TR (2019). Early integration of pastoralism and millet cultivation in Bronze Age Eurasia. Proc. R. Soc. B Biol. Sci..

[122] Lightfoot E, Liu X, Jones MK (2013). Why move starchy cereals? A review of the isotopic evidence for prehistoric millet consumption across Eurasia. World Archaeol..

[123] Miller ARV, Makarewicz CA (2019). Intensification in pastoralist cereal use coincides with the expansion of trans-regional networks in the Eurasian Steppe. Sci. Rep..

[124] Spengler RN, Frachetti MD, Fritz GJ (2013). Ecotopes and herd foraging practices in the steppe/mountain ecotone of Central Asia during the Bronze and Iron Ages. J. Ethnobiol..

[125] Arbuckle BS, Hammer EL (2019). The rise of pastoralism in the ancient near east. J. Archaeol. Res..

[126] Paulette T, Manzanilla L, Rothman MS (2016). Grain, Storage, and State Making in Mesopotamia (3200–2000 BC). Storage in Ancient Complex Societies: Administration, Organization, and Control.

[127] Scott JC (2017). Against the Grain: A Deep History of the Earliest States.

[128] *Prehistoric Archeology along the Zagros Flanks*. (eds. Braidwood, L.S., et al.) (Oriental Institute of the University of Chicago, 1983).

[129] Liu X, Hunt HV, Jones MK (2009). River valleys and foothills: Changing archaeological perceptions of North China’s earliest farms. Antiquity.

[130] Marston JM (2015). Modeling resilience and sustainability in ancient agricultural systems. J. Ethnobiol..

[131] Sołtysiak A, Schutkowski H (2018). Stable isotopic evidence for land use patterns in the Middle Euphrates Valley, Syria. Am. J. Phys. Anthropol..

[132] Marston JM (2011). Archaeological markers of agricultural risk management. J. Anthropol. Archaeol..

[133] Katz O (2010). Rapid phytolith extraction for analysis of phytolith concentrations and assemblages during an excavation: An application at Tell es-Safi/Gath, Israel. J. Archaeol. Sci..

[134] Piperno DR (2006). Phytoliths: A Comprehensive Guide for Archaeologists and Paleoecologists.

[135] Piperno DR (1988). Phytolith Analysis: An Archaeological and Geological Perspective.

[136] *Phytolith Systematics: Emerging Issues*. (eds. Rapp, G. & Mulholland, S.C.) vol. 1 (Springer, 1992).

[137] Twiss PC, Suess E, Smith RM (1969). Morphological classification of grass phytoliths. Soil Sci. Soc. Am. J..

