# Supplementary material for: Phytolith evidence for the pastoral origins of multi-cropping in Mesopotamia (ancient Iraq)
Source: Sci Rep. 2022 Jan 10;12:60. doi: 10.1038/s41598-021-03552-w (PMC8748697; doi:10.1038/s41598-021-03552-w)
Supplement: Supplementary file 1 — Supplementary Information. [file 41598_2021_3552_MOESM1_ESM.pdf]

Supplementary Information for

Phytolith Evidence for the Pastoral Origins of Multi-cropping in Mesopotamia (Ancient Iraq)

Elise Jakoby Laugier[1], Jesse Casana, and Dan Cabanes

[1]**Corresponding author:**
Elise Jakoby Laugier
Elise.J.Laugier.GR@dartmouth.edu

This PDF includes:

Supplementary Text
1 Description of additional contextual data from Khani Masi (SRP046) Trench Y82 2
2 Additional Methods 2

Supplementary Figures
Figure S1. Graph of relative abundances of phytolith morphologies and micro-remain concentrations in each sample arranged by elevation. 3
Figure S2. Phytolith morphometric variables 6
Figure S3. Map illustrating Euclidean distances from Khani Masi to the natural growing range of *Panicum miliaceum*. 9

Supplementary Tables
Table S1. Comparison of sediment sample location, mineralogy, and micro-remain concentrations..........3
Table S2. Sources for archaeological sites in Fig. 1. 4
Table S3. Radiocarbon dates from Khani Masi Area Y82 sequence. 5
Table S4. Bayesian modeled date ranges from Khani Masi Area Y82 sequence. 5
Table S5. Diagnostic criteria of INTERDIGITATING phytolith morphotypes (long cell inflorescence bract/husk of upper lemma and palea) of Broomcorn millet (*P. miliaceum*). 6
Table S6. Selected Panicoideae species with known phytolith inflorescence morphometrics. 7
Table S7. Results of morphometric analysis of INTERDIGITATING phytolith multicellular structures 8
Table S8. Results of Minimum Sample Size Calculations developed by Ball et al. [75,76] 9

Supplementary References 10

Supplementary Text

1 Description of additional contextual data from Khani Masi (SRP046) Trench Y82

Previous geochemical, phytolith, and other micro-remain analysis provides context to the INTERDIGITATING phytolith multicellular structures analyzed in this study [1]. Relevant phytolith and spherulite concentrations are reported in Fig. S1. Table S1 contains the FTIR (Fourier Transform Infrared) spectroscopy assessment of sediment main mineral components, calcite types, and whether clays were altered by heat (burned) (*see Additional methods below*). Bioturbation was carefully avoided during sampling and well-defined horizontal layering indicates contextual integrity and minimal micro-remain translocation.

In general, phytolith preservation from Trench Y82 was good. Phytolith concentrations ranged from 0.4 to 45.9 million/gram of acid insoluble fraction (AIF) (median: 15.9 million/gram of AIF), percent weathered phytoliths was low (<10.1%), percent anatomically connected phytoliths (multicellular structures) was high (median 18.5%), delicate morphologies were present (median: 22.8%), and there were no indications of preservation trends by elevation (Fig. S1) [1,2]. Sediments in trench Y82 alternate between unaltered geogenic fills low in micro-remain concentrations (Facies B) and sediments that are comparatively rich in organic content, micro-remains, and are burned (Facies A). Faecal spherulites were found in high concentrations in sediments with heat altered clays (Facies A: 28.1 ± 10.0 million/g sediment; SD), indicating layers contained discarded dung fuel or burned animal pen accumulations.

The analysis of phytolith morphotypes indicated that the majority of phytoliths are from monocotyledonous grasses (84.7 ± 8.7%) because sedges (Cyperaceae-type) were rare (<2.1%) and no palm phytoliths were identified. Dicotyledonous phytoliths from wood occurred in very low percentages (2.7 ± 1.8%; SD; range: <6.1%). Pooid (C_3; RONDEL, CRENATE, TRAPEZOID) Grass Silica Short Cell Phytoliths (GSSCP) dominated the assemblage (74.2±12.1%; SD) (Fig. S1). Dung-rich layers (Facies A) contained significantly more C_4 GSSCPs (C_3:C_4 ratio, Panicoid (% BILOBATES and POLYLOBATES), and Chloridoid (% SADDLES)) and inflorescence phytoliths (leaf-stem to inflorescence ratio): 0.4 ± 0.2) than fill layers ($p < 0.05$; non-parametric Wilcoxon rank sum test) [1].

2 Additional Methods

FTIR: Sediment mineralogical analysis was performed using a Thermo Scientific Nicolet iS5 FT-IR Spectrometer in the 4000 and 400 cm^{-1} spectral range at 4cm^{-1} resolution. We mixed approximately 1 mg of sample with 80 mg of potassium bromide (KBr) in an agate mill. Main mineral components were determined using the wavelengths of the strongest absorption peaks following [3] and referencing the standards from the Kimmel Center for Archaeological Science, Weizmann Institute of Science. Calcite type was determined using the grinding curve method established by [4]. Clay thermal alteration (heating) was established following [5] as well as the new local thermal alteration references reported in [1].

Phytolith concentrations and morphologies: All samples were assessed for percent organic content using the loss-on-ignition method (550°C for 2 hours) [6]. Subsequently, the same samples were treated with 3N HCl following Albert and Weiner [7] to determine the acid insoluble fraction (AIF). Assessment of phytolith concentrations is in millions per gram of AIF.

Dung Spherulites: Dung spherulites were extracted from untreated sediment and counted following Gur-Arieh et al. [8].

Table S1. Comparison of sediment sample location, mineralogy, and micro-remain concentrations. Main mineral components are ordered by relative peak height in each FTIR spectrum. Data in first seven (of nine) columns was previously reported by [1].

Sample	Location	Phase	Elevation	Main Mineral Components*	Calcite Type	Individual Phytoliths Identified per Slide	Number of Individual Phytoliths Identified in Multicellular structures**	INTERDIGITATING Phytoliths (%)^	Measurable INTERDIGITATING Phytoliths Present
SRP_78	Y82 - disturbed	Plough Zone	190.47	Cl, Ca, Qz	Anthropogenic (Ash)	294	57	1.42	No
SRP_76	Y82	1	190.19	Cl, Gy, Ca	Anthropogenic (Ash)	235	68	0.66	No
SRP_75	Y82	1	190.11	Gy, Cl(a), Ca	n/a (Gypsum)	321	82	0.50	**Yes**
SRP_74	Y82	2	190.05	Ca, Gy, Cl(a)	n/a (Gypsum)	322	110	1.62	No
SRP_73	Y82	2	190.01	Cl(i), Gy, Ca	n/a (Gypsum)	262	91	1.98	**Yes**
SRP_72	Y82	2	189.90	Gy, Cl(a), Ca	Anthropogenic (Ash)	279	240	2.70	No
SRP_69	Y82	4	189.72	Cl(a), Ca, Qz + P	Anthropogenic (Ash)	259	308	1.59	No
SRP_68	Y82	4	189.62	Cl(a), Ca, Qz + P + Gy	Anthropogenic (Ash)	340	145	0.41	No
SRP_67	Y82	4	189.56	Cl(i), Qz, Ca	Geogenic	305	43	2.59	**Yes**
SRP_59	Y82	7	189.02	Cl(a), Qz, Ca + P	Anthropogenic (Ash)	295	157	3.54	**Yes**
SRP_2-20[C]	Control (on-site)	NA	Surface	Cl, Ca, Qz	Geogenic	348	4	0.00	NA

*Ca, calcite; Cl, Clay (a=altered, i = indeterminate); Gy, Gypsum; Qz, quartz, P, phosphate, + minor presence; [C] = Control sample
** Number of phytoliths in anatomical connection (i.e., silica skeletons or articulated multicellular structures)
^ INTERDIGITATING phytoliths in multicellular structures; As percent of total phytoliths (individual phytoliths counted per slide plus number individual phytoliths in anatomical connection)

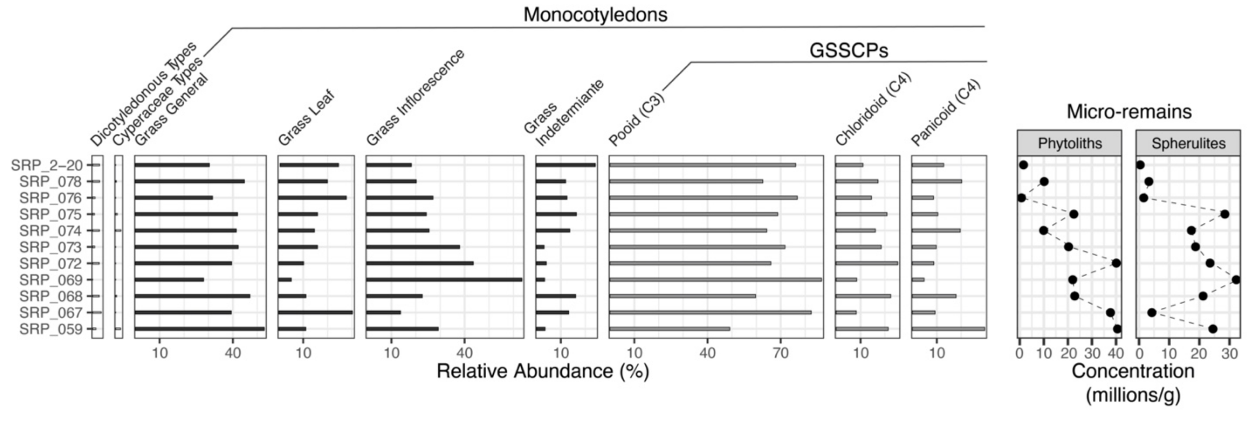

Figure S1. Graph of relative abundances (%) of phytolith morphologies and micro-remain concentrations (million/g of AIF or sediment, respectively) in each sample arranged by elevation. SRP_2-20 is a surface control sample. Grass inflorescences are reported as overall percent and contain both wild and domesticated species. Selected data from [1].

Table S2. Sources for archaeological sites in Fig. 1.

Site No.	Name	Country	Millennium BCE	Dates (ca. BCE)	Reference
1	Golbai Sasan	India	2nd	1500–1113	[9]
2	Raja-Nala-Ka-Tila	India	2nd	1600–1300	[10]
3	Tasbas	Kazakhstan	2nd	1400	[11,12]
4	Begash	Kazakhstan	3rdand1st	2200	[13]
5	Tuzusai	Kazakhstan	1st	410–150	[14]
6	Babor Kot	India	2nd	2000–1700	[15,16]
7	Surkotada	India	3rd-2nd	2500–1700	[17,18]
8	Rojdi	India	3rd-2nd	2500–1700	[17–20]
9	Shortugaï	Afghanistan	2nd	2350–2100	[21]
10	Pirak	Pakistan	2nd	2000–1700	[22]
11	Kyzyltepa	Uzbekistan	1st	6th–4th Cent.	[23]
12	1211/19	Turkmenistan	2nd		[24]
13	Ojakly	Turkmenistan	2nd	1600	[24,25]
14	Gonur Tepe	Turkmenistan	3rd-2nd	2500–1700	[26,27]
15	Tahirbaj Depe	Uzbekistan	1st	650–500	[28,29]
16	Tepe Yahya	Iran	3rd-2nd	2400-1800; 1700-1400	[30,31]
17	Malyan (Anšan)	Iran	3rd-2nd	2400-1800	[32]
18	Gurga Chiya	Iraq	2nd	14th Cent.	[33]
19	Hajar Bin Humeid	Yemen	1st	1300–500	[34]
20	Wadi Yanaiim	Yemen	3rd	2500–2000	[35]
21	Hasanlu	Iran	1st	1300–500	[36,37]
22	Jubabat al-Juruj	Yemen	3rd	2500–2000	[38]
23	Khani Masi	Iraq	2nd	1571–1322	This study
24	Bastam	Iran	1st		[39]
25	Haftavan	Iran	2nd	1900–1500	[40]
26	Nimrud	Iraq	1st	666–626	[41]
27	Jemdet Nasr	Iraq	3rd-2nd		[42–44]
28	Sos Höyük	Turkey	1st		[45]
29	Ziyaret Tepe (Tušhan)	Turkey	1st		[46]
30	Tille Höyük	Turkey	1st	600	[40]
31	Tell Mozan	Syria	2nd	2000–1500	[47]
32	Deir 'Alla	Jordan	1st	ca.1200–500	[48]
33	Kilise Tepe	Turkey	2nd		[49]
34	Tell Sheikh Hamad	Syria	2nd	14th Cent.	[50,51]
35	Gordion	Turkey	1st(Foxtail: 2nd)	800–540 (Foxtail: 1100-900)	[52,53]
36	Kawa	Nubia	1st	800–400	[54]
37	Ukma	Nubia	2nd	2000–1600	[55]
38	Assiros Toumba	Greece	2nd		[56]
39	Kastanas	Greece	2nd		[57,58]

Table S3. Radiocarbon dates from Khani Masi Area Y82 sequence.

Sample	Lab No.	Label Context*	Material	Mass (mg)	Carbon Yield (%)**	$\delta^{13}C$ (‰)	^{14}C Age (uncal. BP)	Reference
R_Date H	AA114857 / X36462	Y82_L20_L4	Charcoal	1.31	54.1	-26.7	3118 ± 29	[1]
R_Date G	AA114856 / X36461	Y82_L18_L2	Charcoal	1.17	54.3	-27.8	3150 ± 27	[1]
R_Date F	AA114854 / X36459	Y82_L9_L6	Charcoal	1.52	59.7	-25.5	3087 ± 27	This Study
R_Date E	AA114866 / X36471	Y82_L9_L3	Charcoal	1.23	45.4	-27.1	3077 ± 30	[1]
R_Date D	AA114851 / X36456	Y82_L7_L3	Charcoal	0.31	43.5	-26.3	3043 ± 41	[1]
R_Date C	AA114853 / X36458	Y82_L9_L2	Charcoal	1.29	54.0	-26.2	3113 ± 30	[1]
R_Date B	AA114852 / X36457	Y82_L9_L1	Charcoal	0.25	NA	-27.2	3041 ± 50	This Study
R_Date A	AA114850 / X36455	Y82_L3_L3	Charcoal	0.15	NA	-26.9	2872 ± 57	This Study

*Excavation Stratigraphic Unit (Area#_Locus#_Lot#)
**Samples with indeterminable carbon yields were marked as NA (not available), by the UArizona AMS Laboratory

Table S4. Bayesian modeled date ranges from Khani Masi Area Y82 sequence.

Sample	Unmodelled (BCE/CE) ±1σ (68.2%)		Unmodelled (BCE/CE) ±2σ (95.4%)		Modelled (BCE/CE) ±1σ (68.2%)		Modelled (BCE/CE) ±2σ (95.4%)		Model Index	Conf-idence
	from	to	from	to	from	to	from	to	A	C
Boundary Start Phase 4					-1468	-1402	-1571	-1322		97.2
**Phase 4**										
R_Date H	-1430	-1313	-1488	-1292	-1428	-1395	-1449	-1321	119.6	99.7
R_Date G	-1492	-1403	-1499	-1320	-1437	-1401	-1491	-1322	111.7	99.6
Boundary End 4 Start 2					-1420	-1374	-1429	-1316		99.7
**Phase 2**										
R_Date F	-1410	-1301	-1421	-1272	-1405	-1335	-1411	-1309	106.3	99.6
R_Date E	-1403	-1296	-1421	-1261	-1402	-1341	-1411	-1308	109.4	99.6
R_Date D	-1387	-1229	-1416	-1133	-1398	-1347	-1410	-1307	102.5	99.6
Boundary End 2 Start 1					-1382	-1318	-1401	-1291		99.5
**Phase 1**										
R_Date C	-1426	-1311	-1446	-1285	-1343	-1296	-1391	-1268	80.9	99.7
R_Date B	-1389	-1224	-1421	-1127	-1351	-1266	-1390	-1221	113.2	99.6
Boundary End 1 Start Plough Zone					-1330	-1205	-1388	-1102		99.4
**Plough Zone**										
R_Date A	-1125	-933	-1220	-903	-1263	-1065	-1377	-943	70.3	99.3
Boundary End Plough Zone					-1254	-998	-1381	-729		95.4

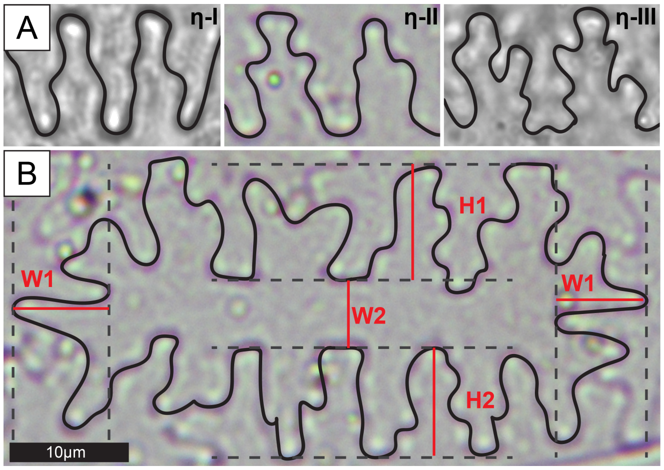

Figure S2. Phytolith morphometric variables. (A) η-type undulation patterns (levels I–III) of epidermal long cells described by Lu et al. [59]. (B) Morphometric measurements used to describe upper lemma and palea (after [59,60]). See Table S5.

Table S5. Diagnostic criteria of the INTERDIGITATING phytolith morphotypes (long cell inflorescence bract/husk of upper lemma and palea) of Broomcorn millet (*P. miliaceum*).

Criteria No.	Long Cell Criteria Description	Metric	Source
1	Papillae	absent	[59,61]
2	Undulation pattern	η-type (levels I-III)	[59]
3	Ending structure	Cross “finger-type”	[59]
4	Ending length (W)	$W = 8.95 \pm 2.02$ μm	[59]
5	Ratio (R) of processes amplitude (H1, H2) to endings (W)[R = W/(H1+H2)/2)]	$R = 0.79 \pm 0.12$ μm	[59,60]
6	Small body width compared to process height: [a]Ratio (H1/W2), [b]Ratio (W2/H1)	>1:1[a]; ≤0.7:1 (or H1/W2=1.43)[b]	[62(a); 60,61(b)]

See Fig. S2 for locations of W, W2, H1, and H2.

Table S6. Selected Panicoideae species with known phytolith inflorescence (INTERDIGITATING) morphometrics.

Species	Common Name	Endemism in Iraq	Endemism Reference	Papillae Present	Undulation (Type - Levels)	Long Cell Ending Type*	W (µm)	R	Process: Body Ratio**	Phytolith Ref.
Sorghum halepense (L.) Pers.	Johnson grass	Native	[63,64]	NA	NA	NA	NA	NA	NA	[65]
Digitaria sanguinalis (L.) Scop.	Crabgrass	Native	[63,64,66,67]	Small conical	Smooth	wavy-type	2.53 ± 0.87	0.23 ± 0.13	R, >1	[62]
Echinochloa colona (L.) Link	Jungle rice	Native	[63,64]	No	β-type, level I	Short [4]	NA	NA	R, >1	[61]
Echinochloa crus-galli (L.) P.Beauv.	Barnyard Millet	Native	[63,64]	No	β-type, levels I–IV	wavy-type	4.86 ± 1.82	0.19 ± 0.09	R, >1	[62,68]
Panicum bisulcatum Thunb.	Japanese Panicgrass	Not Present	[59]	No	η type, level I only	finger-type	NA	NA	R, >1	[59]
Panicum miliaceum L.	Broomcorn Millet	Introduced	[63]	No	η type, levels I–III	finger-type	8.95 ± 2.02	0.79 ± 0.12	R, >1	[59]
Panicum miliaceum subsp. ruderale (Kitag.) Tzvel.	Wild Broomcorn millet	Introduced	[66]	No	η type, levels I–III [2]	NA [5]	NA	NA [5]	R, >1	[69]
Panicum repens L.	Torpedo grass	Native	[63,64]	No	η type, levels I–III	Shorter wavy-type	NA	NA	R, >1	[69]
Panicum turgidum Forssk.	Desert grass	Native (Desert Region)	[63,64,70,71]	NA	NA	NA	NA	NA	NA	[72,73]
Setaria italica (L.) Beauv.	Foxtail millet	Introduced	[63]	Small or no conical	Ω-type, levels I–III	wavy-type	4.37 ± 0.89	0.33 ± 0.11	R, >1	[59]
Setaria pumila (Poir.) Roem. & Schult. [1]	Yellow foxtail millet	Native	[63,64]	Small conical	Ω-type, levels I–III	Smooth	NA	NA	R, >1	[61,73,74]
Setaria verticillata (L.) P. Beauv.	bristly foxtail	Native	[63,64]	Large conical	Ω-type, levels I–II [3]	Smooth	Unmeasurable	NA	R, >1	[62,74]
Setaria viridis (L.) P. Beauv.	Green Foxtail	Native	[63,64]	Large conical	Ω-type, levels I–III	Smooth	Unmeasurable	NA	R, >1	[62,74]
Paspalum dilatatum Poir.	Dallis Grass	Invasive Introduced	[63,64]	Small conical	Ω-type, level I	wavy-type	5.58 ± 2.45	0.58 ± 0.31	R, <1	[62]
Paspalum distichum L.	Knotgrass	Invasive Introduced	[63,64]	NA	NA	NA	NA	NA	NA	

* [59,62]

** R, (H1/W2) >1: Rectangular, small W2 (body); R, (H1/W2) <1: Rectangular, large W2 (body) [62]

[1] synonym: *Setaria helvola* (L.f.) Roem.& Schult.)

[2] η-III less common than *P. miliaceum*. Only one study has determined a distinguishing phytolith metric: *P. miliaceum* tends to have higher percentages of η-III processes than *P. ruderale* [(mean ~10% (range 7-14%) and mean 23% (range 20.7-30.1%), respectively] [69]. Unfortunately, this metric is impossible to determine for most archaeological samples. Here, 6/30 = 20%.

[3] level III rare

[4] similar to *E. crus-galli*

[5] presumably same range as *P. miliaceum*

Table S7. Results of morphometric analysis of INTERDIGITATING phytolith multicellular structures.

**Criteria No.**				**1**	**2**		**3**	**4**		**5**	**6**	
**Lab Photo No.**	**Sample**	**Multicell Code**	**Measured Phytoliths (n)**	**Papillae Present (Y/N)**	**Undulation Pattern**		**Long Cell Ending Type**	**W (µm)**	**Process Height (µm)**	**R**	**Process: Body Ratio**	**Criteria Met (n)**
					**Type**	**Level**						
S67_02	S67	1	2	N	η	III	Finger	9.90 ± 1.78	14.24 ± 0.63	0.69 ± 0.09	1.93 ± 0.13	6
S73_01	S73	1	3	N	η	I–II	Wave	7.37 ± 0.86	11.15 ± 1.48	0.67 ± 0.14	1.91 ± 0.37	5
S75_01	S75	1	4	N	η	I–II	Finger/Wave	8.43 ± 0.29	12.27 ± 1.01	0.69 ± 0.05	2.22 ± 0.32	6
S59_01	S59	1	2	N	η	III	Finger	9.51 ± 1.49	11.86 ± 0.46	0.80 ± 0.09	1.78 ± 0.47	6
S59_03	S59	2	2	N	η	II–III	Wave	7.47 ± 0.25	9.65 ± 0.84	0.78 ± 0.04	2.00 ± 0.03	5
S59_08	S59	3	10	N	η	I	Finger/Wave	7.18 ± 0.76	11.16 ± 0.54	*0.64 ± 0.08*	1.42 ± 0.15	5
S59_09	S59	4	3	N	η	I	Finger/Wave	*6.65 ± 1.62*	10.77 ± 0.66	*0.61 ± 0.13*	1.46 ± 0.12	4
S59_12	S59	5	1	N	η	II–III	Finger	8.45	11.23	0.75	1.17	6
S59_13	S59	6	4	N	η	II	Finger/Wave	8.03 ± 0.33	12.18 ± 0.91	*0.66 ± 0.06*	1.83 ± 0.22	5
S59_14	S59	7	1	N	η	I–II	Finger/Wave	7.72 ± 0.43	11.44	0.67	1.86	6
S59_04	S59	8	5	N	η	II–III	Finger/Wave	7.00 ± 1.13	11.09 ± 0.46	*0.63 ± 0.11*	2.25 ± 0.20	5
LSC59_04	S59	9	2	N	η	I	Finger	10.91 ± 0.00	14.23 ± 0.53	0.77 ± 0.03	1.89 ± 0.05	6
LSC59_08	S59	10	1	N	η	III	Finger	9.49	12.62	0.75	1.7	6
LSC59_24	S59	11	1	N	η	I	Finger/Wave	8.81	12.87	0.68	2.3	6
LSC59_25	S59	12	1	N	η	I–II	Finger	8.60	13.93	*0.62*	1.75	5
LSC59_32	S59	13	2	N	η	II	Wave	*6.85 ± 0.13*	10.90 ± 1.88	*0.64 ± 0.10*	1.88 ± 0.18	3
LSC59_33	S59	14	2	N	η	II	Finger	8.81 ± 0.23	11.85 ± 1.04	0.75 ± 0.09	1.50 ± 0.08	6
LSC59_38	S59	15	1	N	η	III	Finger	9.25	12.50	0.74	1.62	6
LSC59_39	S59	16	2	N	η	I	Finger/Wave	8.44 ± 0.37	11.70 ± 0.19	0.72 ± 0.02	1.08 ± 0.04	6
LSC59_42	S59	17	6	N	η	I	Finger/Wave	*6.35 ± 0.32*	9.43 ± 1.07	0.68 ± 0.07	1.33 ± 0.19	5
LSC59_44	S59	18	4	N	η	II–III	Finger	8.38 ± 0.78	11.83 ± 1.31	0.71 ± 0.05	2.02 ± 0.36	6
LSC59_45	S59	19	7	N	η	I–II	Finger	7.63 ± 0.87	10.81 ± 0.52	0.71 ± 0.06	1.59 ± 0.12	6
LSC59_47	S59	20	2	N	η	I	Finger	7.68 ± 0.04	11.20 ± 0.24	0.69 ± 0.02	1.58 ± 0.15	6
LSC59_48	S59	21	5	N	η	I	Finger/Wave	8.23 ± 0.70	11.69 ± 0.69	0.70 ± 0.04	1.59 ± 0.21	6
LSC59_53	S59	22	3	N	η	I–II	Finger/Wave	8.12 ± 0.16	10.95 ± 0.25	0.74 ± 0.01	1.44 ± 0.11	6
LSC59_55	S59	23	4	N	η	I	Finger/Wave	*6.47 ± 0.80*	8.08 ± 0.60	0.81 ± 0.14	1.28 ± 0.11	5
LSC59_60	S59	24	3	N	η	I–II	Finger/Wave	7.28 ± 0.32	9.85 ± 1.12	0.74 ± 0.07	1.93 ± 0.35	6
LSC59_61	S59	25	1	N	η	I–II	Finger	8.20	13.23	*0.62*	1.74	5
LSC59_65	S59	26	2	N	η	I	Finger/Wave	6.95 ± 2.28	9.22 ± 0.19	0.75 ± 0.23	2.21 ± 0.64	6
LSC59_67	S59	27	4	N	η	II	Finger	9.66 ± 0.80	11.90 ± 0.42	0.81 ± 0.08	1.92 ± 0.14	6

Values are reported as average ± one standard deviation.

In italics: average values below one standard deviation values for *P. miliaceum* Lu et al. [59].

Table S8. Results of Minimum Sample Size Calculations developed by Ball et al. [75,76]

	W (µm)	Process Height (µm)	R	W2 (µm)	
Overall Average ± Standard Deviation*	8.13 ± 1.10	11.53 ± 1.43	0.71 ± 0.06	6.93 ± 1.41	
	Theoretical Minimum Counts Required per Measurement				Actual number counted in this study
Multicellular Phytolith Count	12.12	10.08	4.31	27.33	30
Individual Phytolith Count	16.23	11.02	10.2	25.46	90

* Values are reported as averages ± one standard deviation.

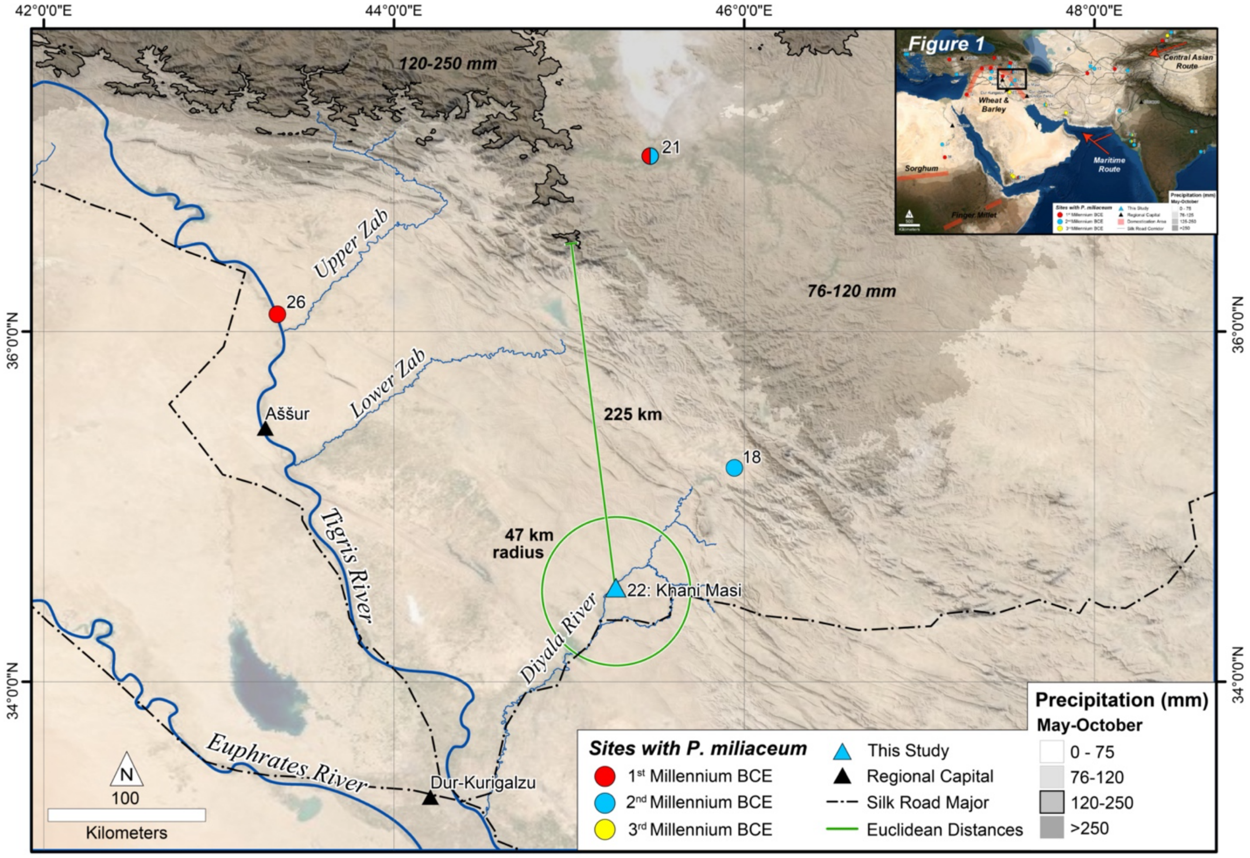

Figure S3. Map illustrating Euclidean distances from Khani Masi to the natural growing range of *Panicum miliaceum* (outlined dark gray area; >120mm precipitation May-October) [37,77]. The 47 km radius circle (green) indicates the maximum one-way travel distance for plant matter consumed by sheep/goats based on average herd speeds and digestion times [78]. The green line indicates the Euclidean distance to nearest area in which *P. miliaceum* can be cultivated with precipitation. Dashed lines show later, reconstructed silk road corridors (after [79]). This figure was generated in Esri's ArcGIS 10.6.1 (http://www.esri.com/software/arcgis) using a digital elevation hillshade derived from 7.5-arc-second Global Multi-resolution Terrain Elevation Data 2010 (GMTED2010) courtesy of the USGS and Esri World Imagery (Sources: Esri, DigitalGlobe, GeoEye, i-cubed, USDA FSA, USGS, AEX, Getmapping, Aerogrid, IGN, IGP, swisstopo, and the GIS User Community).

Supplementary References

1. Laugier, E. J., Casana, J., Glatz, C., Sameen, S. M. & Cabanes, D. Reconstructing agro-pastoral practice in the Mesopotamian-Zagros borderlands: Insights from phytolith and FTIR analysis of a dung-rich deposit. *J. Archaeol. Sci. Rep.* **38**, 103106 (2021).
2. Madella, M. & Lancelotti, C. Taphonomy and phytoliths: A user manual. *Quat. Int.* **275**, 76–83 (2012).
3. Weiner, S. *Microarchaeology: beyond the visible archaeological record*. (Cambridge University Press, 2010).
4. Regev, L., Poduska, K. M., Addadi, L., Weiner, S. & Boaretto, E. Distinguishing between calcites formed by different mechanisms using infrared spectrometry: archaeological applications. *J. Archaeol. Sci.* **37**, 3022–3029 (2010).
5. Berna, F. *et al.* Sediments exposed to high temperatures: reconstructing pyrotechnological processes in Late Bronze and Iron Age Strata at Tel Dor (Israel). *J. Archaeol. Sci.* **34**, 358–373 (2007).
6. Dean, W. E. Determination of carbonate and organic matter in calcareous sediments and sedimentary rocks by loss on ignition; comparison with other methods. *J. Sediment. Res.* **44**, 242–248 (1974).
7. Albert, R. M. & Weiner, S. Study of phytoliths in prehistoric ash layers from Kebara and Tabun caves using a quantitative approach. in *Phytoliths: applications in earth sciences and human history* (eds. Meunier, J. D. & Colin, F.) 251–266 (A.A. Balkema Publishers, 2001).
8. Gur-Arieh, S., Mintz, E., Boaretto, E. & Shahack-Gross, R. An ethnoarchaeological study of cooking installations in rural Uzbekistan: development of a new method for identification of fuel sources. *J. Archaeol. Sci.* **40**, 4331–4347 (2013).
9. Kingwell-Banham, E. J. Early rice agriculture in South Asia. Identifying cultivation systems using archaeobotany. *Doctoral thesis, UCL (University College London).* (UCL (University College London), 2015).
10. Saraswat, K. S. Agricultural background of the early farming communities in the Middle Ganga Plain. *Pragdhara* **15**, 145–177 (2005).
11. Doumani, P. N. *et al.* Burial ritual, agriculture, and craft production among Bronze Age pastoralists at Tasbas (Kazakhstan). *Archaeol. Res. Asia* **1–2**, 17–32 (2015).
12. Spengler, R. N., Frachetti, M. D. & Doumani, P. N. Late Bronze Age agriculture at Tasbas in the Dzhungar Mountains of eastern Kazakhstan. *Quat. Int.* **348**, 147–157 (2014).
13. Frachetti, M. D., Spengler, R. N., Fritz, G. J. & Mar'yashev, A. N. Earliest direct evidence for broomcorn millet and wheat in the central Eurasian steppe region. *Antiquity* **84**, 993–1010 (2010).
14. Spengler, R. N., Chang, C. & Tourtellotte, P. A. Agricultural production in the Central Asian mountains: Tuzusai, Kazakhstan (410–150 b.c.). *J. Field Archaeol.* **38**, 68–85 (2013).
15. Reddy, S. N. *Discerning Palates of the Past: An Ethnoarchaeological Study of Crop Cultivation and Plant Usage in India*. (International Monographs in Prehistory, 2003).
16. Reddy, S. N. Plant usage and subsistence modeling: An ethnoarchaeological approach to the Late Harappan of northwest India. (The University of Wisconsin - Madison, 1994).
17. Liu, X. *et al.* From ecological opportunism to multi-cropping: Mapping food globalisation in prehistory. *Quat. Sci. Rev.* **206**, 21–28 (2019).
18. Pokharia, A. K., Kharakwal, J. S. & Srivastava, A. Archaeobotanical evidence of millets in the Indian subcontinent with some observations on their role in the Indus civilization. *J. Archaeol. Sci.* **42**, 442–455 (2014).
19. *Food economy of the Harappans*. (ed. Possehl, G.L.) (Oxford and IBH, 1982).

20. Weber, S. A. Millets in South Asia: Rojdi as a case study. in *South Asian Archaeology 1987* (ed. Taddie, M.) 333–348 (Istituto Italiano per il Medio ed Estremo Oriente, 1990).
21. Willcox, G. Carbonised plant remains from Shortugai, Afghanistan. in *New light on early farming: recent developments in palaeoethnobotany* (ed. Renfrew, J. M.) 139–153 (Edinburgh University Press, 1991).
22. Costantini, L. Plant remains at Pirak. in *Fouilles de Pirak* (eds. Jarrige, J.-F. & Santoni, M.) 326–333 (Diffusion de Boccard, 1979).
23. Wu, X., Miller, N. F. & Crabtree, P. Agro-Pastoral Strategies and Food Production on the Achaemenid Frontier in Central Asia: A Case Study of Kyzyltepa in Southern Uzbekistan. *Iran* **53**, 93–117 (2015).
24. Rouse, L. M. & Cerasetti, B. Ojakly: A Late Bronze Age mobile pastoralist site in the Murghab Region, Turkmenistan. *J. Field Archaeol.* **39**, 32–50 (2014).
25. Spengler, R. N., Cerasetti, B., Tengberg, M., Cattani, M. & Rouse, L. M. Agriculturalists and pastoralists: Bronze Age economy of the Murghab alluvial fan, southern Central Asia. *Veg. Hist. Archaeobotany* **23**, 805–820 (2014).
26. Anthony, D. W. *The Horse, the Wheel, and Language: How Bronze-Age Riders from the Eurasian Steppes Shaped the Modern World*. (Princeton University Press, 2007).
27. Bakels, C. C. The contents of ceramic vessels in the Bactria-Margiana Archaeological Complex, Turkmenistan. *Electron. J. Vedic Stud.* **9**, 49–52 (2003).
28. Nesbitt, M. Archaeobotanical research in the Merv Oasis. In: The International Merv Project Preliminary Report on the Second Season (1993). *Iran* **32**, 53–75 (1994).
29. Neef, R. Die Pflanzenreste. in *Die Skythenzeitliche Fürstenkurgan Aržan 2 in Tuva* (eds. Čugonov, K. V., Parzinger, H. & Nagler, A.) 242–248 (Philipp Von Zabern, 2010).
30. Costantini, L. & Biasini, L. C. Agriculture in Baluchistan between the 7th and the 3rd Millennium B. C. *Newsl. Baluchistan Stud.* **2**, 16–37 (1985).
31. Lamberg-Karlovsky, C. C. *Excavations at Tepe Yahya, Iran, 1967-1975: the early periods*. (Peabody Museum of Archeology and Ethnology, Harvard University, 1986).
32. Miller, N. F. Economy and environment of Malyan, a third millennium BC urban center in southern Iran. (The University of Michigan, 1982).
33. Wengrow, D. *et al.* Gurga Chiya and Tepe Marani: New Excavations in the Shahrizor Plain, Iraqi Kurdistan. *Iraq* **78**, 253–284 (2016).
34. Fuller, D. Q. & Boivin, N. Crops, cattle and commensals across the Indian Ocean. Current and Potential Archaeobiological Evidence. *Études Océan Indien* **42–43**, 13–46 (2009).
35. Ekstrom, H. & Edens, C. M. Prehistoric agriculture in highland Yemen: New results from Dhamar. *Bull. Am. Inst. Yemeni Stud.* **45**, 23–35 (2003).
36. Costantini, L. Hasanlu Plant Remains. in *(unpublished list. Hasanlu archive)* (University of Pennsylvania Museum., n.d.).
37. Miller, N. F., Spengler, R. N. & Frachetti, M. Millet cultivation across Eurasia: Origins, spread, and the influence of seasonal climate. *The Holocene* **26**, 1566–1575 (2016).
38. Costantini, L. Ecology and farming of the protohistoric communities in the central Yemeni highlands. in *The Bronze Age culture of hawlan at-Tiyal and al-Hada (Republic of Yemen)* (ed. de Maigret, A.) 187–204 (IsMEO, 1990).
39. Hopf, M. Pflanzenresten. in *Bastam II, Ausgrabungen in den Urartaischen Anlagen 1977–1978* (ed. Kleiss, W.) 263–318 (Deutsches Archäologisches Institut, 1989).
40. Nesbitt, M. & Summers, G. D. Some recent Discoveries of Millet (*Panicum Miliaceum* L. and *Setaria italica* (L.) P. Beauv.) at Excavations in Turkey and Iran. *Anatol. Stud.* **38**, 85–97 (1988).

41. Helbaek, H. The Plant Remains from Nimrud. in *Nimrud and its Remains, Volume II* (ed. Mallowan, M. E. L.) 613–620 (Collins, 1966).
42. Field, H. Ancient Wheat and Barley from Kish, Mesopotamia. *Am. Anthropol.* **34**, 303–309 (1932).
43. Helbaek, H. The Paleoethnobotany of the Near East and Europe. in *Prehistoric investigations in Iraqi Kurdistan* (eds. Braidwood, R. J. & Howe, B.) 99–118 (University of Chicago Press, 1960).
44. Jacobsen, T. *Salinity and irrigation agriculture in antiquity: Diyala Basin archaeological projects: report on essential results, 1957-58*. (Undena Publ., 1982).
45. Longford, C., Drinnan, A. & Sagona, A. Archaeobotany of Sos Höyük, northeast Turkey. in *New Directions in Archaeological Science* (eds. Fairbairn, A., O'Connor, S. & Marwick, B.) 121–136 (ANU Press, 2009). doi:10.22459/TA28.02.2009.09.
46. Rosenzweig, M. S. 2 Assessing the Politics of Neo-Assyrian Agriculture. *Archeol. Pap. Am. Anthropol. Assoc.* **29**, 30–50 (2018).
47. Riehl, S. Erste ergebnisse der archäobotanischen untersuchungen in der zentralen oberstadt von Tall Mozan/Urkeš im rahmen der DOG-IIMAS-Kooperation. *Mitteilungen Dtsch. Orient-Ges. Zu Berl.* **132**, 229–238 (2000).
48. Neef, R. Plants. in *Picking up the threads: A continuing review of excavations at Deir Alla, Jordan* (eds. van der Kooij, G. & Ibrahim, M. H.) 30–37 (University of Leiden, Archaeological Centre, 1989).
49. Bending, J. & Colledge, S. The Archaeobotanical Assemblages. in *Excavations at Kilise Tepe: 1994-98; from Bronze Age to Byzantium in western Cilicia, Volume 1* (eds. Postgate, N. & Thomas, D.) 583–596 (McDonald Institute for Archaeological Research, 2007).
50. van Zeist, W. Comments on Plant Cultivation at Two Sites on the Khabur, North-Eastern Syria. in *Umwelt und Subsistenze der assyrischen Stadt Dur-Katlimmu am unteren Habur* (ed. Kühne, H.) vol. 8 133–147 (Harrassowitz, 2008).
51. van Zeist, W. Comments on plant cultivation at two sites on the Khabur, North-eastern Syria. in *Reports on archaeobotanical studies in the Old World.* (ed. van Zeist, W.) 33–60 (2003).
52. Marston, J. M. *Agricultural Sustainability and Environmental Change at Ancient Gordion: Gordion Special Studies 8*. (University of Pennsylvania Press, 2017).
53. Miller, N. F. *Botanical Aspects of Environment and Economy at Gordion, Turkey*. (University of Pennsylvania Museum, 2010).
54. Fuller, D. Q. Early Kushite Agriculture: Archaeobotanical Evidence from Kawa. *Sudan Nubia* **8**, 70–74 (2004).
55. Van Zeist, W. The plant remains. in *Le Cimetière Kermaïque d'Ukma Ouest* (ed. Vila, A.) 247–255 (CNRS, 1987).
56. Jones, G., Wardle, K., Halstead, P. & Wardle, D. Crop Storage at Assiros. *Sci. Am.* **254**, 96–103 (1986).
57. Kroll, H. Bronze Age and Iron Age agriculture in Kastanas, Macedonia. in *Plants and Ancient Man: Studies in Palaeoethnobotany: Proceedings of the Sixth Symposium of the International Work Group for Palaeoethnobotany, Groningen, 30 May-3 June 1983* (eds. van Zeist, W. & Casparie, W. A.) 243–246 (Balkema, 1984).
58. Kroll, H. *Kastanas: Ausgrabungen in einem Siedlungshügel der Bronze- und Eisenzeit Makedoniens, 1975-1979.* (Volker Spiess, 1983).
59. Lu, H. *et al.* Phytoliths Analysis for the Discrimination of Foxtail Millet (*Setaria italica*) and Common Millet (*Panicum miliaceum*). *PLOS ONE* **4**, e4448 (2009).
60. Zhang, J., Lu, H., Wu, N., Yang, X. & Diao, X. Phytolith Analysis for Differentiating between Foxtail Millet (*Setaria italica*) and Green Foxtail (*Setaria viridis*). *PLOS ONE* **6**, e19726 (2011).

61. Weisskopf, A. R. & Lee, G.-A. Phytolith identification criteria for foxtail and broomcorn millets: a new approach to calculating crop ratios. *Archaeol. Anthropol. Sci.* **8**, 29–42 (2016).
62. Ge, Y., Lu, H., Zhang, J., Wang, C. & Gao, X. Phytoliths in Inflorescence Bracts: Preliminary Results of an Investigation on Common Panicoideae Plants in China. *Front. Plant Sci.* **10**, (2020).
63. Rudov, A., Mashkour, M., Djamali, M. & Akhani, H. A Review of C4 Plants in Southwest Asia: An Ecological, Geographical and Taxonomical Analysis of a Region with High Diversity of C4 Eudicots. *Front. Plant Sci.* **11**, (2020).
64. Hanelt, P. *Mansfeld's encyclopedia of agricultural and horticultural crops*. (Springer, 2001).
65. Tripathi, D. K., Mishra, S., Chauhan, D. K., Tiwari, S. P. & Kumar, C. Typological and Frequency Based Study of Opaline Silica (Phytolith) Deposition in Two Common Indian Sorghum L. Species. *Proc. Natl. Acad. Sci. India Sect. B Biol. Sci.* **83**, 97–104 (2013).
66. Nesbitt, M. *Identification Guide for Near Eastern Grass Seeds*. (Institute of Archaeology, UCL, 2006).
67. *Small millets in global agriculture. Proceedings of the First International Small Millets Workshop Bangalore, India, October 29-November 2, 1986*. (eds. Seetharam, A., Riley, Ken W. & Harinarayana, G.) (Oxford & IBH Publishing Co., 1989).
68. Ge, Y. *et al.* Phytolith analysis for the identification of barnyard millet (*Echinochloa* sp.) and its implications. *Archaeol. Anthropol. Sci.* **10**, 61–73 (2018).
69. Zhang, J. *et al.* Phytolith analysis for differentiating between broomcorn millet (*Panicum miliaceum*) and its weed/feral type (*Panicum ruderale*). *Sci. Rep.* **8**, 1–9 (2018).
70. Bor, N. L. Gramineae. in *Flora of Iraq, Vol. 9. Gramineae.* (eds. Townsend, C. C., Guest, E. & al-Rawi, A.) vol. 9 (Ministry of Agriculture & Agrarian Reform, 1968).
71. Ghazanfar, S. A. & McDaniel, T. Floras of the Middle East: A Quantitative Analysis and Biogeography of the Flora of Iraq. *Edinb. J. Bot.* **73**, 1–24 (2016).
72. Radomski, K. U. & Neumann, K. Grasses and grinding stones: inflorescence phytoliths from tvioclern West African Poaceae and Archaeological Stone artefacts. in *Windows on the African Past Current: Approaches to African Archaeobotany* (eds. Fahmy, A. G., Kahlheber, S. & D'Andrea, A. C.) 153–166 (Africa Magna Verlag, 2011).
73. Weisskopf, A. R. *Millets, rice and farmers: phytoliths as indicators of agricultural, social and ecological change in Neolithic and Bronze Age central China*. (British Archaeological Reports, 2014).
74. Bhat, M. A., Shakoor, S. A., Badgal, P. & Soodan, A. S. Taxonomic Demarcation of *Setaria pumila* (Poir.) Roem. & Schult., *S. verticillata* (L.) P. Beauv., and *S. viridis* (L.) P. Beauv. (Cenchrinae, Paniceae, Panicoideae, Poaceae) From Phytolith Signatures. *Front. Plant Sci.* **9**, (2018).
75. Ball, T. B., Vrydaghs, L., Van Den Hauwe, I., Manwaring, J. & De Langhe, E. Differentiating banana phytoliths: wild and edible *Musa acuminata* and *Musa balbisiana*. *J. Archaeol. Sci.* **33**, 1228–1236 (2006).
76. Ball, T. B. *et al.* Morphometric analysis of phytoliths: recommendations towards standardization from the International Committee for Phytolith Morphometrics. *J. Archaeol. Sci.* **68**, 106–111 (2016).
77. Fick, S. E. & Hijmans, R. J. WorldClim 2: new 1-km spatial resolution climate surfaces for global land areas. *Int. J. Climatol.* **37**, 4302–4315 (2017).
78. Dunseth, Z. C. *et al.* Archaeobotanical proxies and archaeological interpretation: A comparative study of phytoliths, pollen and seeds in dung pellets and refuse deposits at Early Islamic Shivta, Negev, Israel. *Quat. Sci. Rev.* **211**, 166–185 (2019).
79. Williams, T. *The Silk Roads: An ICOMOS Thematic Study*. (ICOMOS, 2014).
